# Generalized metric for broadband flat lens performance comparison

**DOI:** 10.1515/nanoph-2022-0196

**Published:** 2022-07-14

**Authors:** Jacob Engelberg, Uriel Levy

**Affiliations:** Department of Applied Physics, The Center for Nanoscience and Nanotechnology, The Hebrew University, Jerusalem 91904, Israel

**Keywords:** broadband, diffractive lens, flat lens, metalens, performance

## Abstract

A plethora of metalenses and diffractive lenses (“flat lenses”) have been demonstrated over the years. Recently, attempts have been made to stretch their performance envelope, particularly in the direction of wide-band achromatic performance. While achromatic behavior has been demonstrated, showing an actual improvement in imaging performance relative to conventional (non-chromatically corrected) flat lenses has remained a major challenge. The reasons for this are use of inappropriate performance metrics, lack of comparison to a baseline conventional design, and lack of a performance metric that combines signal-to-noise ratio and resolution. An additional problem is that different published flat lens designs use different first order parameters, so they cannot be compared. In this work we present an overall performance metric that will allow comparison of different types of flat lenses, even if their first order optical parameters are not the same. We apply this metric to several published achromatic flat lens designs and compare them to the equivalent conventional flat lens, which we consider as the lower bound for achromatic flat lens performance. We found that the performance of the achromatic flat lenses studied does not surpass that of a conventional diffractive lens. Use of this metric paves the way for future developments in the field of achromatic flat lenses, which will display proven progress.

## 1 Introduction

Flat lenses, which can be implemented as diffractive lenses or metalenses, hold a great promise for miniaturization and economical mass production of optical systems by replacement of conventional refractive lenses [1–3]. However, the resolution of conventional non-chromatically corrected flat lenses, which we will call “conventional flat lenses”, is limited by their strong chromatic aberration [4]. This drawback motivates recent research on the development of achromatic flat lenses [5–7]. Unfortunately, the achromatization usually comes at the expense of reduced efficiency, lens power, and field-of-view (FOV).

**Figure 1:**
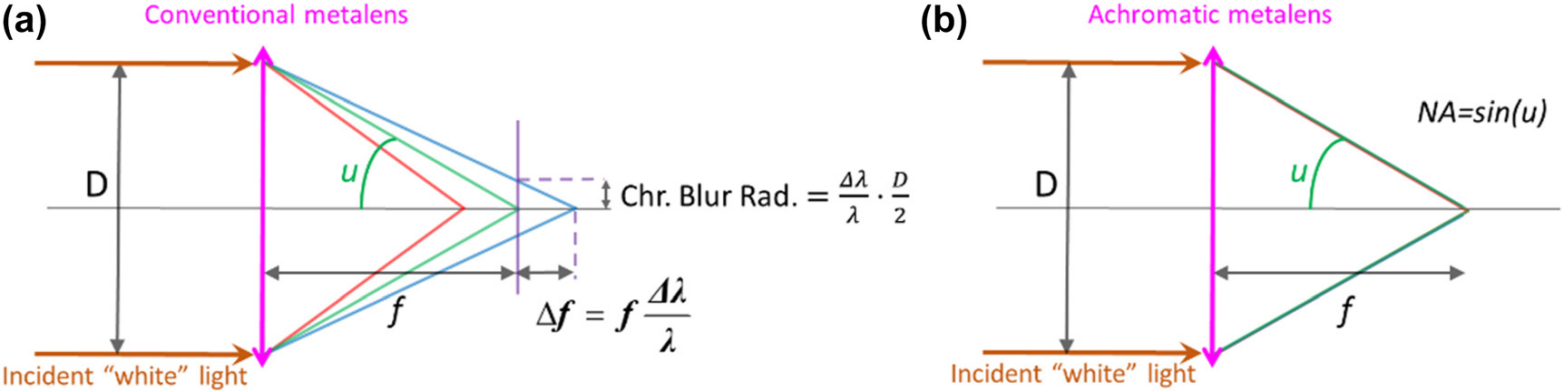
Sketch showing ray paths, basic parameters (lens diameter D, focal length *f*, and numerical aperture NA) and chromatic aberration (longitudinal chromatic aberration Δ*f*, and chromatic blur radius) for (a) conventional versus, (b) achromatic, diffractive lens.

Implementing an achromatic flat lens, be it either an achromatic diffractive lens or an achromatic metalens, is challenging. Nevertheless, there have been several demonstrations of achromatic metalenses of different types such as dispersion engineered, spatial multiplexed, and extended-depth-of-focus [3, 7], [8], [9], [10], [11]. Recently there have also been demonstrations of achromatic diffractive lenses, leveraging the capabilities of extended phase depth and advanced optimization techniques [6]. A sketch of conventional versus achromatic flat lens behavior, in terms of ray optics, is shown in Figure 1 [12].

The demonstrated achromatic flat lenses have various first-order parameters, shown in Figure 1: Aperture diameter *D*, focal length *f*, numerical aperture $\text{NA}=\sin\left(u\right)=\sin\left[atan\left(D/2f\right)\right]$ , central operating-wavelength (CWL) *λ*_*0*_, spectral range Δ*λ*, and sometimes a certain field-of-view (FOV, not drawn). The challenge in assessing the contribution of a new implementation or design lies in the difficulty to determine the level of progress beyond the state of the art (the demonstrated lenses are mostly generic and not intended for a specific application, so the innovation cannot usually be determined based on functionality). This causes research in the field of flat lenses to lack a clear path of progress.

As of today, the performance metrics used by most authors are the focusing efficiency and full-width half-maximum (FWHM) of a light spot in the image plane. The definitions of focusing efficiency and diffraction efficiency, and the relation between them, are discussed in Section 1 of the Supplementary. In this paper, when simulating efficiency, we used classic diffraction efficiency, i.e., the fraction of the light incident on the lens aperture that goes to the design diffraction order. Efficiency represents the signal level but does not account for the background illumination, caused by spurious transmitted diffraction orders, that contributes to noise in an imaging system. Therefore, even if the efficiency is known, the effect of the flat lens on the signal-to-noise ratio (SNR) cannot be uniquely determined. Even worse, the FWHM of the measured spot (which is generally compared to the FWHM of the diffraction limited spot) which is supposed to represent the resolution, is in fact not an effective measure of resolution, as explained in Section 2 of the Supplementary, as well as in ref. [13].

An additional problem with the methods used to evaluate flat lenses is that the signal/efficiency and resolution parameters are treated separately, although they are clearly coupled [14, 15]. For a conventional flat lens used for imaging applications with broadband scene illumination, there is a tradeoff between signal and resolution, based on the spectral bandwidth used. A narrow bandwidth will provide good resolution (small chromatic aberration) at the price of low signal and SNR (since not many photons from the broadband scene illumination have frequencies within the limited bandwidth of the system), while a broad bandwidth will give high signal/SNR at the expense of low resolution due to aberrations. Such a low-resolution/high-SNR image can be converted to a high-resolution/low-SNR image by performing a deconvolution via digital image processing. A similar tradeoff exists for the relative aperture (i.e. the NA or the *F#*, defined as *F# = f/D*) of the conventional flat lens [12]. These tradeoffs are also relevant to achromatic flat lenses since their achievable level of resolution and efficiency is related to the level of chromatic aberration before correction (which in turn depends on spectral range and aperture). More importantly for our analysis, achromatic metalenses generally achieve improved resolution at the expense of efficiency, since larger metalens nano-structure height and refractive index contrast increase the achievable resolution, but tend to decrease the efficiency [16]. To summarize, an overall performance metric that accounts for both resolution and SNR is needed for proper evaluation of flat lens performance. This is the main purpose of our manuscript.

Perhaps the greatest problem preventing understanding of the quality of achromatic designs is that they are seldom compared to the equivalent conventional design. A true comparison should, of course, use a metric that combines resolution and SNR. However, even at the more basic level of resolution only, a proper comparison is rarely made.

In most publications the quantitative evaluation of achromatic flat lenses has been done at discrete wavelengths. While this may be appropriate for some non-imaging applications (such as relay optics in a WDM communication system), the most common application for achromatic lenses is imaging over a continuous spectrum. In this paper we focus on such applications, so our metric and examples are tailored to lenses operating over a continuous spectral range. However, the metric we shall present can be adapted to non-imaging applications as well.

Finally, it would be very useful to have a performance metric that will allow comparison not only of lenses with the same first order parameters, but also of lenses with different parameters. This will allow us to compare between published designs and assess the relative level of achievement, even if they do not have the same first-order parameters. As will be shown, by considering both SNR and resolution we can formulate such a metric.

Following recent research, in which the upper limits of achromatic flat lens performance were explored [16, 17], in this paper we compare such achromatic flat lenses to the benchmark in the field, set by the equivalent conventional flat lens. Our purpose is to answer the following question: Do current versions of achromatic flat lenses perform better than conventional flat lenses for broadband imaging applications? To answer this question, we combine resolution and efficiency into a single metric that allows us to compare overall achromatic flat lens performance to the benchmark of conventional flat lens performance. We also compare different achromatic flat lens designs, to see which gives the best results. An important question that arises is how much room is left between the upper limit and the benchmark, i.e., can achromatic flat lenses make a significant contribution relative to conventional flat lenses? This is discussed in [17], where it is found that not much room is indeed left. However, the upper bounds apply only to a certain class of achromatic metalenses and achromatic diffractive lenses (single layer dispersion-engineered metalens and single surface multilevel diffractive lens, respectively), so these limits could be surpassed by using other approaches. *The metric presented in this paper can allow us to evaluate the level of contribution of such future designs.*

In Section 2 we introduce our overall performance metric (OPM), for comparison of flat lenses with identical first order parameters. In Section 3 we develop the extended overall performance metric (EOPM) for comparison of flat lenses with different parameters. In Section 4 we expand these metrics to color imaging applications and to applications where the field-of-view is significant (imaging applications). In Section 5 we apply these metrics to several published achromatic flat lens designs and compare them to equivalent conventional diffractive lens designs. Analyses of additional published achromatic flat lens designs for color imaging applications are presented in Section 8 of the Supplementary. Finally, in Section 6 we draw our conclusions.

## 2 Overall performance metric

In the following we describe an overall performance metric for imaging applications based on average signal-to-noise ratio (ASNR), as defined by Eq. (1) [18]. Here, the signal-to noise ratio (SNR) refers to zero (or low) spatial frequency SNR, where the modulation transfer function (MTF) takes a value of 1. We assume a shot noise limited system, which is a “white” noise, i.e., equal amplitude at all spatial frequencies. The shot noise arises from the statistical nature of the arrival of the photons and the creation of free electrons in the sensor pixels. The noise level is defined as the standard deviation of the temporally varying signal level, and in the case of shot noise it is proportional to the square root of the number of photons/free electrons. The noise is therefore a temporal noise, but since it occurs independently for each pixel it gives rise to a spatial noise (which is temporally varying). The spatial and temporal standard deviations are equal due to ergodicity [19, 20].

If we produce an image of a large dark area on a white background, the noise is the standard deviation of the pixel gray-level values in the white image area, and the signal is the difference between the average white and dark gray-level values. To obtain the SNR at higher spatial frequencies, we multiply this SNR by the MTF of the system, to account for the signal attenuation at these spatial frequencies. The noise, however, is assumed to be constant over all frequencies, as mentioned. Therefore, the SNR at any given spatial frequency is equal to the zero-frequency SNR multiplied by the MTF at that frequency. To obtain the ASNR one needs to average over all the spatial frequencies, as shown in Eq. (1). This expression is similar to what is obtained from an information theory approach, only that here we omit the log function [21]. We will revisit this in Section 4, where we discuss extended FOV systems.

(1) $$\begin{array}{cc} \text{ASNR} & =\int_{0}^{\nu_{nq}}\text{SNR}\cdot \text{MTF}\left(\nu \right)dv/\nu_{nq},\\ \nu_{nq} & =\frac{1}{2\cdot \text{pixel}\text{\_}\text{pitch}} \end{array}$$

The zero-frequency SNR in Eq. (1) is frequency independent, so it can go outside the integral. The integral limits, going from zero to Nyquist frequency, *ν*_*nq*_, are relevant when working with a specific camera. However, if we want to make an evaluation of the metalens regardless of the camera being used, we can take this limit to be the lens MTF cutoff (i.e., we assume the camera has small enough pixels such that the metalens rather than the camera is limiting the resolution). In this case the area under the lens MTF, relative to the area under the diffraction limited MTF, is equal to the one-dimensional version of the Strehl ratio – see Section 2 of Supplementary. Since the diffraction limited MTF is determined by the first-order lens parameters (NA and spectrum), we can use the Strehl ratio as a single number measure of resolution, instead of the MTF, if we compare lenses with the same parameters.

The SNR in the shot-noise limited case is proportional to the square root of the number of photons reaching a single camera pixel [12]. The absolute number of photons is dependent on many variables, but if we compare two flat lens systems with the same ambient and system parameters (target illumination, NA, spectral range, camera pixel size and integration time) we can say that the number of photons is proportional only to the flat lens efficiency [22].

The last statement is true if the incident light that does not reach the desired focal point is reflected or absorbed. However, in most realistic imaging applications a significant amount of light is lost to transmitted spurious diffraction orders, causing veiling glare (VG) [23, 24]. We denote the total transmitted fraction of the incident light (going to all transmitted diffraction orders) as *T*, and the fraction of incident light that goes to the desired order of diffraction (usually the first order) as *η*. The SNR is then proportional to $\eta /\sqrt{T}$ , since the total number of photons contributing to noise is proportional to *T*, while the signal is proportional to *η* (Alternatively, we can reach the same expression by saying that the overall SNR is proportional to $\sqrt{T}$ , when we initially consider all the light as signal. When we then take into account that our signal is only part of the total transmitted light, usually the first order of diffraction, the spurious diffraction orders reduce the contrast, i.e., the un-normalized MTF, by a factor of *η*/*T* [23]. When multiplied, this gives the same $\eta /\sqrt{T}$ factor). See Section 3 of the Supplementary for discussion of how to measure this factor.

Based on the above considerations, we can define an overall performance metric (OPM) that is proportional to the ASNR according to Eq. (2).

(2) $$\text{OPM}=\frac{\eta }{\sqrt{T}}\cdot \text{Strehl}$$

Since in most publications no information is given regarding the overall transmission *T*, we are forced to take the optimistic assumption that there is no VG, i.e., *T* = *η*, in our analyses of published results shown in the following sections. In such a case, one obtains $\text{OPM}=\sqrt{\eta }\cdot \text{Strehl}$ . However, it must be remembered that these are optimistic results. The other extreme case would be to assume *T* = 1. In such a case the difference between transmission and efficiency is attributed to VG, and the OPM is given by OPM = *η* · Strehl which is always smaller than the previous expression.

The OPM equals 1 in an ideal case, and 0 in the worst case. The values for *η* and *T* used in Eq. (2) should be a weighted average over the spectral range, based on the detector spectral responsivity. It is important to note that the above merit function is only effective for comparing metalenses of different types (such as conventional diffractive lens/metalens vs. achromatic diffractive lens/achromatic metalens) that share identical first-order parameters (relative aperture, focal length, spectral range, field-of-view). If one wants to quantitatively compare lenses of different parameters, the extended metric presented next should be used.

## 3 Extended overall performance metric

To devise a metric that can be used to compare the performance of lenses with different first order parameters, we use the following guidelines: (a) The signal of a scene illuminated by a broad spectrum is proportional to the spectral range Δλ that is allowed to pass through to the detector [12]. We elect to introduce a relative spectral range factor of $\sqrt{\Delta \lambda /\lambda }$ into our metric, thus keeping the merit function unitless, but limiting it to comparison of lenses operating around the same CWL (the radical is because the SNR is proportional to the square root of the number of photons). (b) The larger the relative aperture the higher the SNR. The signal is proportional to the NA^2^ [25, 26], so the SNR will be proportional to the NA. (c) As stated in the previous section, use of the Strehl ratio instead of the area under the MTF is only justified if we are comparing lenses of the same parameters. If we are comparing lenses with different parameters, we must multiply the Strehl ratio by the area under diffraction limited MTF, to obtain the area under the aberrated MTF. We can approximate this area by using the diffraction limited MTF for a square aperture, which is a triangle having an area of *ν*_co_/2 = NA/*λ*, where *ν*_co_ is the diffraction limit MTF cutoff (the factor between the areas under MTF of round aperture to MTF of square aperture is 8/(3*π*)) [26, 27]. In effect, to compare lenses of different parameters, what we want is a merit function that is related not to the average SNR but rather to the integrated SNR (over spatial frequencies). Thus, unlike the expression for ASNR (Eq. (1)), one should not divide by the frequency range. (d) The Strehl ratio and diffraction limit cutoff frequency only account for the image space resolution. However, what is really of interest is the object space resolution. For an imaging lens viewing a distant object the magnification is proportional to the lens focal length *f*, so we add this factor to our merit function (for other imaging applications, such as a microscope objective, the focal length alone does not determine the magnification, since it depends on the tube lens focal length. However, a short focal length generally means a small field-of-view and short working distance, so a larger *f* has an advantage there too).

Implementing the above-mentioned guidelines, we now arrive at Eq. (3), which defines the extended OPM (EOPM) which allows us to compare between lenses having different first order parameters, limited only to the same CWL.

(3) $$\text{EOPM}=f\cdot \text{NA}\cdot \sqrt{\frac{\Delta \lambda }{\lambda }}\cdot \frac{\text{NA}}{\lambda }\cdot \text{OPM}$$

To simplify Eq. (3), we can use the Fresnel number (FN), given by Eq. (4) [28]. The FN is approximately equal to the maximum (unwrapped) phase induced in the wavefront by the lens, modulo *π*, so the higher the FN the greater the “work” the lens is doing [29]. Substituting Eq. (4) into Eq. (3) we obtain Eq. (5).

(4) $$\text{FN}\approx \frac{f{\cdot \text{NA}}^{2}}{\lambda }$$

(5) $$\text{EOPM}=\text{FN}\sqrt{\frac{\Delta \lambda }{\lambda }}\cdot \text{OPM}=\text{FN}\sqrt{\frac{\Delta \lambda }{\lambda }}\cdot \frac{\eta }{\sqrt{T}}\cdot \text{Strehl}$$

As opposed to the OPM, which gives a value between 0 and 1, the EOPM is not limited by an upper bound. As previously mentioned, the EOPM is limited to comparing lenses with the same central operating wavelength (CWL). However, this is not a severe limitation, since it is generally not relevant to compare lenses operating at different spectral ranges, because of the different operating conditions (different scene illumination, available materials for optics and detector etc.).

How should Δλ be defined? While for a Gaussian distribution one may simply use the FWHM as the measure of spectral width, this is not the case for a general spectral distribution. The more general choice is to use the standard deviation of the spectral response when viewed as a probability distribution, multiplied by a constant. The definition of the constant is flexible, as long it is used consistently, since the EOPM is relative. For the simulations presented in this paper, we chose the constant to be $2\sqrt{3}$ , since this results in the full width for the case of a uniform (“Rect” function) spectral distribution.

Not surprisingly, the various parameters that go into the EOPM not only determine the performance, but also the design and manufacturing challenge. The larger the relative spectral range ( Δλ/λ ) the more challenging the achromatic design is, since the chromatic blur spot is proportional to this ratio [12]. The larger the relative aperture (high NA or lower *F#*) the more challenging the design, since the diffraction limited spot size gets smaller, and the geometrical aberrations become more significant. The larger the lens (i.e., the larger the absolute value of the focal length/aperture) the more challenging the design (for a specific NA), since geometrical aberrations scale with lens dimensions while the diffraction limit does not [30, 31].

This significance of the focal length is an important point, since many publications boast of a high-NA achromatic flat lens, but since the focal length of the lens is very short (often on the order of tens of microns), the FN is small, and the lens is not useful. In fact, for a thin plano-convex refractive lens the sagitta of the convex surface is related to the FN by $\text{sag}=\lambda \frac{\text{FN}}{2\left(n-1\right)}$ . Therefore, if the FN is on the order of 1–10, the refractive lens “thickness”, i.e., the sagitta, is on the order of the wavelength, so it too can also be considered flat. From a manufacturing point of view, it may require different methods since it cannot be composed of discrete levels. Still, in certain cases it may be relevant to compare achromatic flat lens performance to that of the refractive equivalent.

**Figure 2:**
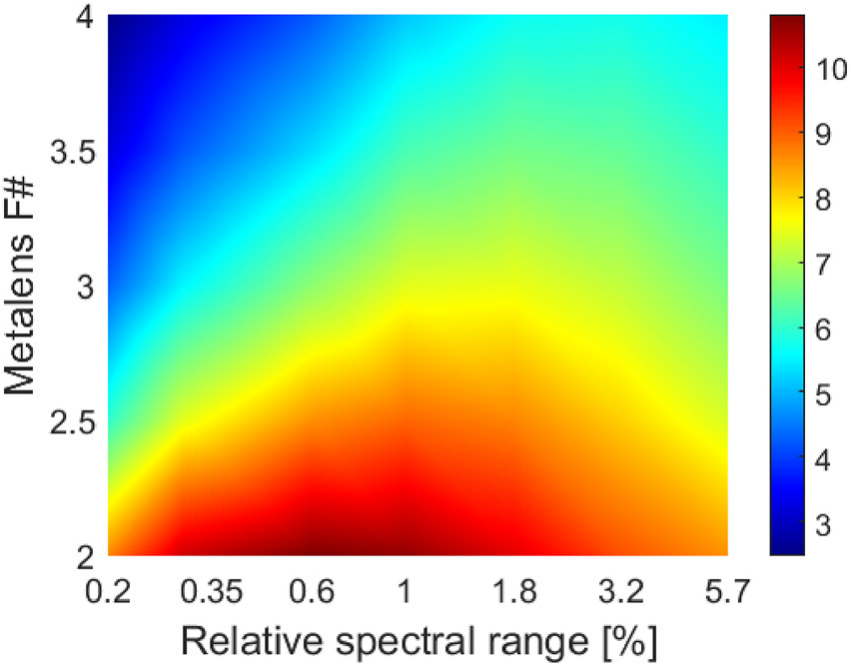
EOPM for a conventional diffractive lens as a function of *F*# and relative spectral bandwidth Δλ/λ. For a given *F*#, one can determine the optimal operating waveband.

Note that the EOPM can be used not only to evaluate the performance of a design, but also to optimize its parameters. In a previous paper we showed how such optimization can be performed based on the ASNR metric [12]. By maximizing the EOPM we can perform a similar optimization, under the assumption that the resolution is limited by the optics rather than by the camera. For example, the maximum EOPM can give an indication of which aperture to choose given the spectral range, and vice versa. This method can be applied to both conventional and achromatic flat lenses. In Figure 2 we present the EOPM calculated for a simple binary (two-phase levels) conventional diffractive lens, with a focal length of 5.2 mm operating around a central wavelength of 550 nm (the general shape of the graph is similar to the ASNR graphs shown in [12, 18]). The absolute value of the EOPM will change for a multilevel conventional diffractive lens, since the efficiency will improve, but the shape of the graph will remain the same.

Based on Figure 2, for a given *F*# and central wavelength, there is an optimal spectral bandwidth that should be used to maximize the conventional diffractive lens performance. However, it seems that the lower the *F*# the better the performance. What limits the *F*# on the low end? There are of course manufacturing/spatial sampling considerations, since for low *F*# one needs a very small diffractive period. However, even if we consider ideal manufacturing capabilities, there is a limit derived from field-of-view (FOV) considerations. The lower the *F*# the more difficult it becomes to obtain good performance over a reasonable FOV. Even for a system designed to operate on-axis only, one must account for this, because of tolerance issues. The way to do this is discussed in Section 4.

As can be seen from Figure 2, the EOPM varies even for a given flat lens technology. Although the EOPM can be used to compare lenses made using various technologies, it also includes the effects of the system parameters. Therefore, one cannot compare the EOPM for two lenses of identical parameters and conclude that a specific technology is always superior to the other. To compare two competing technologies, one must find the optimum EOPM for each of them, while allowing the first order lens parameters to vary over the available/practical range (e.g., if we want to make a camera lens with a specific FOV, we can vary the aperture and the wavelength range, as in Figure 2). If relevant, the EOPM*fov* described in section 4 should be used. The technology that produces a better EOPM is the preferred one for the task, at least in terms of optical performance (practical considerations, such as manufacturing cost must be considered separately).

## 4 Expansions of performance metrics

In this paper we focus on comparing achromatic flat lens to conventional flat lens performance for the case of broadband imaging applications with a small FOV. However, the OPM and EOPM metrics can be adapted and used for other applications as well. For example, for the case of a non-imaging system, such as a WDM fiber coupling lens, one may replace the 1D Strehl ratio with an overlap integral (for single-mode fiber), or with a parameter related to encircled energy (for multi-mode fiber). If the operation can be limited to discrete wavelengths, the performance should be evaluated and averaged over these wavelengths only.

The metrics can also be applied to systems operating quasi-monochromatically, where chromatic aberration is not an issue, and no added energy is gained by opening the spectral range. In this case, the $\sqrt{\Delta \lambda /\lambda }$ factor can be omitted.

A particularly interesting application is color imaging. For a color imaging system that has three separate channels, red (R), green (G), and blue (B), we can extend the above-mentioned discussion and use an average EOPM of the channels (with Δλ/λ referring to the spectral range of each channel) to obtain the quality of the intensity component of the image (equal to R + G + B, where each letter represents the relevant gray-level image). However, when performing color imaging there is another performance parameter of interest: color fidelity. To obtain good color fidelity, it is not sufficient to have good average EOPM, rather one must have good EOPM for each individual channel. Therefore, for color imaging we suggest the metric of Eq. (6), which not only takes into account the average EOPM but also the standard deviation of the three channels, in such a way that it decreases with the increase in the standard deviation of the EOPM. The correction term (in the square brackets) multiplying the average EOPM was chosen such that it will be zero when the EOPM of two out of three channels is zero (meaning we have no color information), and 1 when all three EOPMs are equal (for derivation see Section 4 of Supplementary). If one is comparing color imaging lenses of identical first order parameters, the EOPM in Eq. (6) can be replaced with the OPM.

(6) $$\text{EOPMcolor}=\text{avg}\left(\text{EOPM}\right)\left[1-\frac{\text{std}\left(\text{EOPM}\right)}{\sqrt{3}\text{avg}\left(\text{EOPM}\right)}\right]$$

Another factor that affects color fidelity is the shape of the spectral response of the flat lens [32, 33], but that is beyond the scope of this paper. Here we assume that the spectral response of the lens is sufficiently broad so that the overall response is dominated by the camera, which has assumedly been designed to provide good color fidelity. Of course, it is possible to formulate other performance metrics for a color system, for example by replacing the 1 in the brackets with a 2, thus allotting some value to a system that gives a black and white image and giving extra credit for displaying color.

An additional important application is systems with a significant FOV. While in this paper we will apply the merit functions on-axis only, they can be extended to consider the FOV. The question is how does one account for the benefit of a larger FOV system? Defining a merit function (EPOMfov) for an optical system with a field-of-view is a bit tricky, since as mentioned in section 2, it is now necessary to apply a log function to the integrand in Eq. (1), to be consistent with information theory. The theory was originally developed for temporally varying electrical signals [34–36], but was shortly thereafter applied to spatial images [21, 37, 38]. As explained by Hartley [34], the need for the log function arises from the fact that although the number of permutations possible in an image of *g* gray-levels and *p* pixels is equal to *g* ^*p*^, the objective amount of information increases only linearly with *p*. The information content is therefore better represented by $\log\left(g^{p}\right)=p\cdot \text{log}\left(g\right)$ . This is also in line with the logarithmic sensitivity of the human eye. Use of a base 2 logarithm is convenient since it represents the number of bits necessary to transmit the information, although for our purposes any base can be used.

We therefore suggest the EOPMfov figure of merit given by Eq. (7), where *A* is the image area, based on the development in Section 5 of the Supplementary. This figure of merit is more limited than the on-axis EOPM, in that the camera parameters and scene illumination must be considered in calculating the SNR, see Eq. S11. Lenses must be compared over the same image area *A*, but this does not limit the generality. If the MTF varies significantly over the FOV, the FOV must be broken up into isoplanatic patches (areas over which the MTF is nearly constant). In such a case the figure of merit should be calculated for each area separately, and then summed [37]. Note that the focal length factor is not included in this merit function. It is no longer necessary since the image area fulfills a similar function in representing the system scaling.

(7) $$\text{EOPMfov}=A\overset{\nu_{\text{co}}}{\underset{-\nu_{\text{co}}}{\iint }}\log\left[1+{\text{SNR}}^{2}\cdot {\text{MTF}}^{2}\left(\nu_{x},\nu_{y}\right)\right]d\nu_{x}d\nu_{y}$$

## 5 Analysis of published achromatic flat lens results

To demonstrate the utility of our proposed metric, we apply it to several published achromatic flat lens designs. Each time we will compare the achromatic flat lens to the baseline, i.e., the equivalent (same first order optical parameters) conventional diffractive lens. This will allow us to evaluate the contribution (if any) of the achromatic design to the improvement of the optical performance for the chosen parameters. The more appropriate comparison would be to a conventional flat lens of the same type as the achromatic flat lens. For example, in the case of a dispersion engineered metalens one should compare to a conventional metalens using the same materials and manufacturing method. However, since we are evaluating designs using several different technologies, it is simpler for us to compare to a single baseline design type.

We will first present case studies of two single waveband designs, using dispersion engineered metalens and inverse-designed multilevel diffractive lens technologies. We will then present two triple-waveband (R, G, and B) color imaging case-studies. For the color designs we will analyze two publications using methods of spatial multiplexing and extended depth-of-focus (EDOF). We relegate to Section 8 of the Supplementary two additional case studies of achromatic flat lenses using the same technology as the single waveband examples, but now applied to color imaging.

### 5.1 Dispersion engineered achromatic metalens

We begin with three achromatic metalens designs for the short-wave infrared (SWIR) spectral range presented in [5], based on dispersion engineered nanostructures. The optical parameters for the designs are summarized in the top rows of Table 1. We included the Airy radius and the chromatic aberration radius in the table, since these can be calculated using simple formulas, and allow a quick check of the level of chromatic aberration relative to the diffraction limit. Obviously, if the chromatic aberration is not much larger than the Airy radius, there is less need to correct the chromatic aberration. EFL in the table stands for the effective focal length of the lens (which is the same as the focal length but was used historically when describing complex lens system as opposed to thin lenses).

**Table 1:** Comparison of achromatic metalenses (AMLs) of [5] to equivalent conventional diffractive lenses (CDLs).

Design	M1B		M2		M3	
λmin [µm]	1.2		1.2		1.2	
λmax [µm]	1.65		1.65		1.4	
NA	0.24		0.13		0.88	
EFL [µm]	200		800		30	
Dia. [µm]	100		210		111	
Fresnel no.	8		10		51	
Airy rad [µm]	3.6		6.7		0.9	
Chr. rad [µm]	7.8		16.6		4.3	
	**AML**	**CDL**	**AML**	**CDL**	**AML**	**CDL**
Efficiency	0.35	0.78	0.35	0.87	?	0.05
2D Strehl	0.85	0.57	0.85	0.61	?	0.16
1D Strehl	∼0.93	0.72	∼0.93	0.71	?	0.35
OPM	0.55	0.64	0.55	0.66		0.08
EOPM	2.6	3.0	3	3.6		1.5

**Figure 3:**
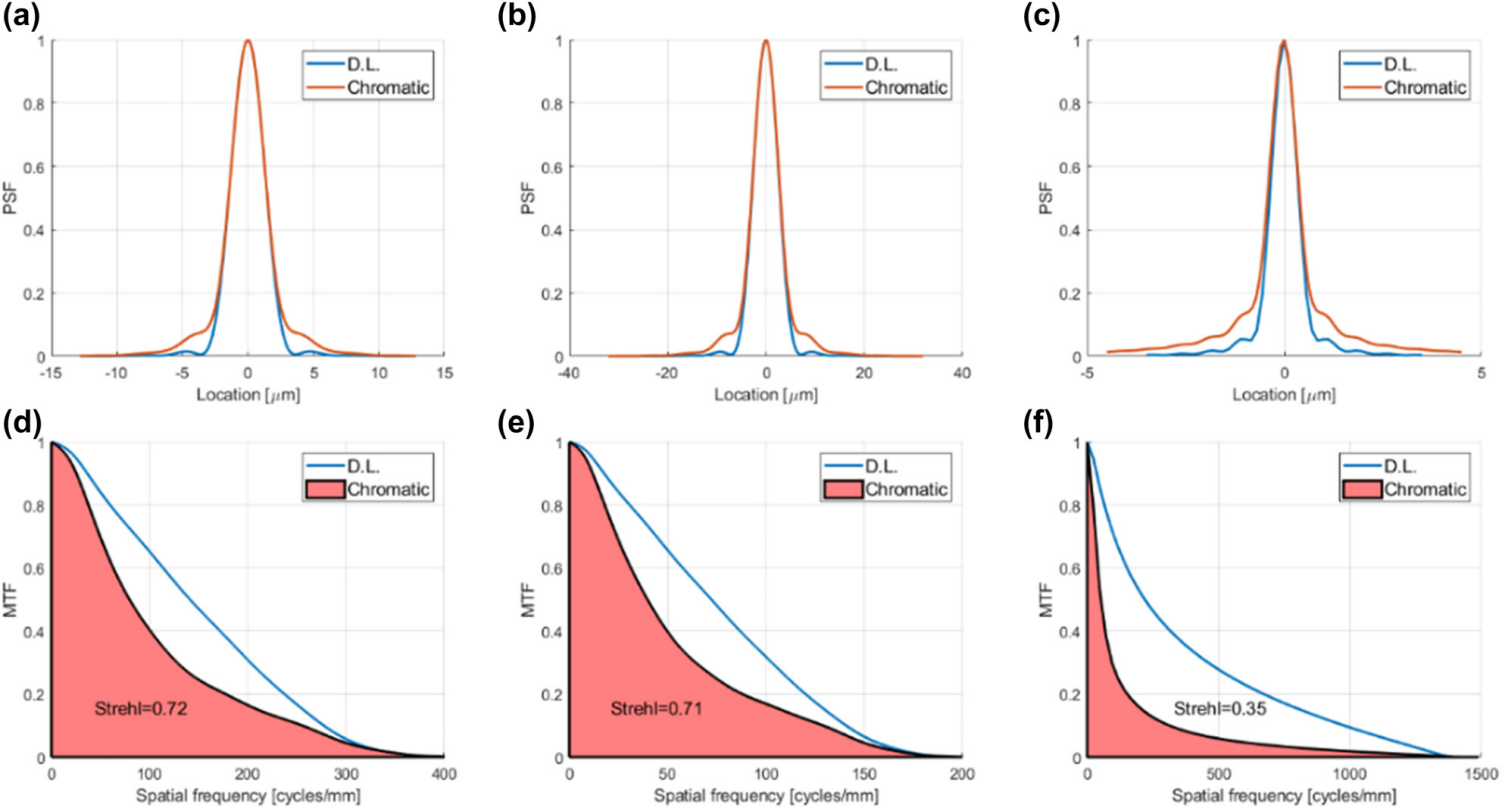
PSFs, MTFs, and 1D Strehl ratio of conventional diffractive lens designs equivalent to achromatic metalens designs of [5], compared to the diffraction limit. (a) PSF cross section of conventional diffractive lens equivalent to achromatic metalens design M1B compared to diffraction limited PSF (b) The same for design M2 (c) The same for design M3 (d) MTF of conventional diffractive lens equivalent to M1B compared to diffraction limited MTF, showing the 1D Strehl ratio, which is the ratio of shaded pink area to the area under the D.L. graph. (e) The same for design M2. (f) The same for design M3.

The published measured achromatic metalens performance is summarized under the column titled “AML” in the bottom rows of Table 1. This performance is compared to the simulated performance of a classic 8 phase-level conventional diffractive lens (It would have been more appropriate to use simulated performance data for the metalens, since we are comparing to simulation, but none were given in this publication. However, for our conventional diffractive lens designs it is reasonable to assume performance close to theory, since there exists a well-established manufacturing technology [4, 39]). The optical performance parameters (PSF, MTF, and Strehl ratio) for the conventional diffractive lenses were calculated using commercial optical design software (see Section 6 of Supplementary for details). The results are shown in Figure 3, and summarized in Table 1, under the column titled ’CDL’. The efficiency of the conventional diffractive lens was calculated analytically based on Eq. 12 of [40] (accounting for Fresnel reflection, number of phase levels and the shadow effect) and the spectral range (see Section 7 of Supplementary for details). Uniform spectral response over the wavelength range was assumed. In principle we could have compared to a conventional (non-chromatically corrected) metalens, rather than to a diffractive lens. The reason we chose the diffractive lens option is because the efficiency can be calculated analytically.

It can be seen from Figure 3 that for the first two designs (M1B and M2) the chromatic aberration of the equivalent conventional diffractive lens is not so large, so the resolution is not far from the diffraction limit, albeit not diffraction limited (the 2D Strehl is less than 0.8, which is generally accepted as the threshold for diffraction limited performance). While the achromatic metalens improves the resolution (higher Strehl ratio), the price paid in reduced efficiency makes the overall imaging performance worse than the conventional diffractive lens (lower OPM). In [5] the 2D Strehl ratio was given, and not the 1D. To compute our metrics, we estimated the 1D Strehl to be the average between the 2D Strehl and 1 (the 1D Strehl is almost always higher than the 2D, since the 2D Strehl overemphasizes the high frequencies, which naturally have lower MTF – see Section 2 of Supplementary). In our experience the average between the 2D Strehl and 1 gives an optimistic value for the 1D Strehl.

**Figure 4:**
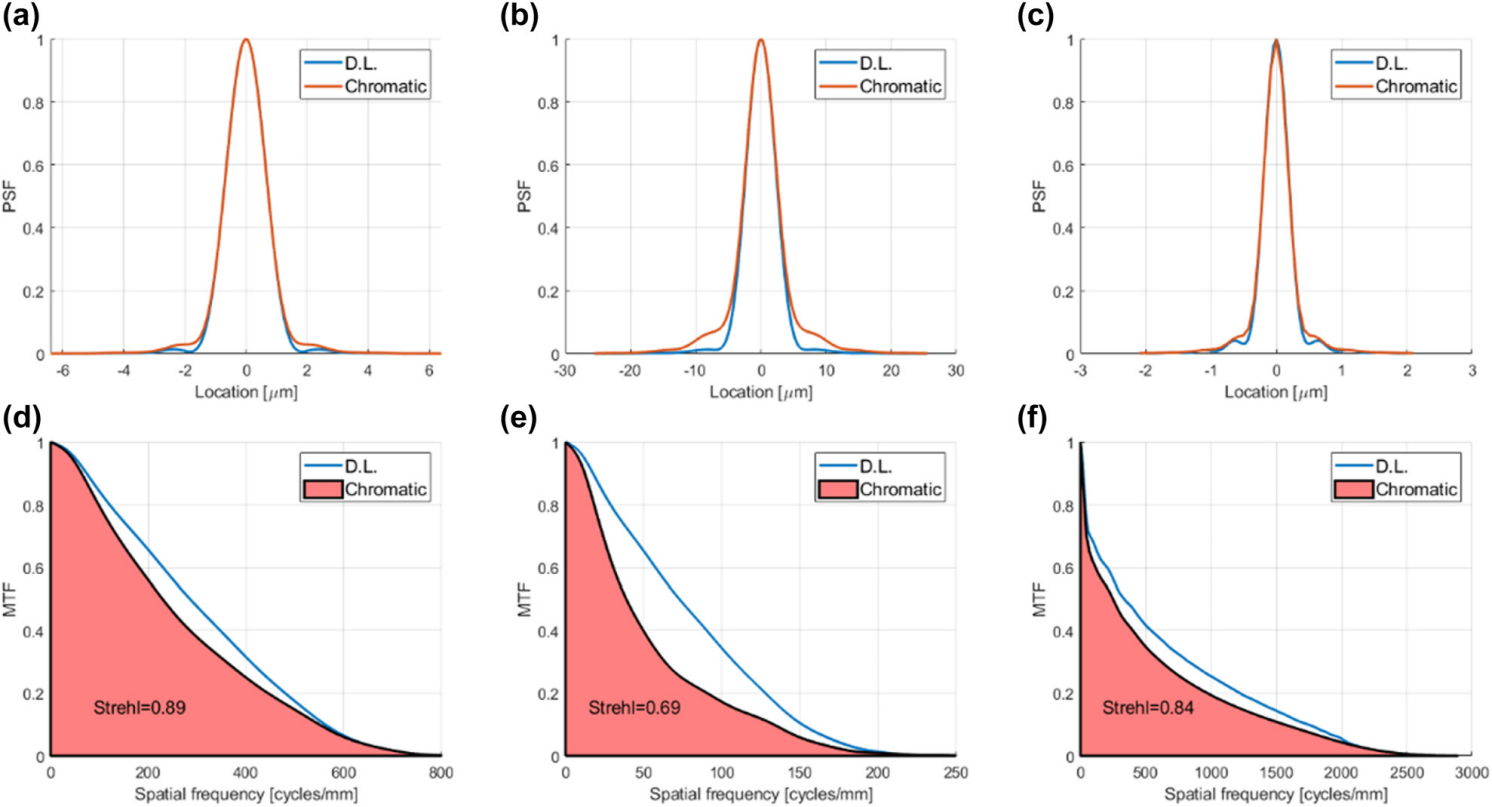
PSFs, MTFs, and 1D Strehl ratio of conventional diffractive lens designs equivalent to broadband achromatic metalens and achromatic diffractive lens designs of [44], compared to the diffraction limit. (a) PSF cross section of conventional diffractive lens equivalent to Design 1 of [44] (b) The same for Design 2 (c) The same for Design 3 (d) MTF of conventional diffractive lens equivalent to Design 1 of [44] (e) The same for Design 2 (f) The same for Design 3.

The third design (M3) is a high-NA design with more significant chromatic aberration, i.e., there is greater potential for improvement by using an achromatic metalens. Unfortunately, the authors did not present resolution data for this design, claiming that Strehl ratio is not applicable to high-NA lenses. We beg to differ with this claim. If there is a measured or simulated PSF, and a diffraction limited PSF can be calculated, there is a Strehl ratio [27, 41, 42]. It is true that the calculation is more complicated in the high-NA case, as explained in Section 6 of Supplementary, giving rise to the non-typical “belly” in the shape of the diffraction limited MTF (Figures 3(f) and 4(f)). The only case when the Strehl ratio is not relevant is when the scalar approximation breaks down, i.e. the lens aperture is on the order of the wavelength, since in such a case the diffraction limited PSF cannot be calculated [43].

The efficiency of design M3 was also not measured, so we cannot compare to the conventional diffractive lens efficiency. The low conventional diffractive lens efficiency is caused primarily by poor phase sampling near the edge of the lens (we assumed a minimum feature size of 1 µm) and includes the shadowing effect. Both effects can be partially overcome by a metalens, so a conventional metalens might give even better performance.

### 5.2 Inverse-designed multilevel diffractive lens

We now move on to a paper by Banerji et al. that compares the performance of metalens designs to equivalent inverse-designed multilevel diffractive lenses [44]. Specifically, we apply our metric to the three broadband designs that were presented in [44]. It can be seen from Figure 4 and Table 2 that for all three designs the conventional diffractive lens resolution is not far from the diffraction limit. The quoted achromatic metalenses give only a small improvement in resolution over the conventional diffractive lenses, and the price paid in efficiency is large, so the overall performance is worse. The achromatic diffractive lenses designed by Banerji et al. improve upon the achromatic metalenses in terms of efficiency, but no real indication is given of the resolution performance, since the authors use the FWHM metric. The numbers marked in blue in the table show the 1D Strehl ratio that would be needed for the achromatic diffractive lens designs to exceed the performance of the conventional diffractive lenses.

**Table 2:** Comparison of achromatic diffractive lenses (ADLs) of [44] and achromatic metalenses (AMLs) quoted therein to equivalent conventional diffractive lenses (CDLs).

Design	1			2			3		
λmin [µm]	0.47			3			0.56		
λmax [µm]	0.67			5			0.8		
NA	0.2			0.36			0.81		
EFL [µm]	63			155			2		
Dia. [µm]	25.7			119.6			5.5		
Fresnel no.	4.6			5.6			4.1		
Airy rad [µm]	1.74			6.78			0.51		
Chr. rad [µm]	2.3			15			0.5		
DL FWHM	1.48			5.26			0.37		
	**AML [8]**	**ADL**	**CDL**	**AML [45]**	**ADL**	**CDL**	**AML [46]**	**ADL**	**CDL**
Efficiency	0.5	0.81	0.67	0.7	0.86	0.54	0.69	0.7	0.08
FWHM	1.5	1.31	1.47	5	5.49	5.46	0.39	0.39	0.36
2D Strehl	0.92	?	0.82	0.38	?	0.5	?	?	0.62
1D Strehl	∼0.96	0.81	0.89	∼0.69	0.55	0.69	0.29	0.29	0.84
OPM	0.68		0.73	0.32		0.51			0.24
EOPM	1.83		1.96	1.25		2.00			0.59

It should be noted that for the “achromatic metalens” of Design 3 no claim is made that the design is achromatic [46], i.e., it is actually a conventional metalens that is simply used over a broad spectral band, with good results. It can be seen from Figure 4(f) why this is, since a conventional diffractive lens with identical parameters also gives near diffraction limited results over this range. It should also be noted that for the small aperture of this design (5.5 micron) our simulated conventional diffractive lens performance based on the scalar approximation may not be accurate. Such a design can be accurately simulated using FDTD or RCWA software. At any rate, lenses of such small dimensions are generally not relevant to imaging applications, which is the focus of this work.

The previous designs were analyzed assuming they were not intended for color imaging. For the case of color imaging the contribution of achromatic flat lenses becomes greater. This is because if the lens is optimally focused for the green (G) channel, the red (R) and blue (B) channels will be quite defocused. In the G channel, the central in-focus wavelengths give rise to a sharp peak in the PSF, which in turn creates a long “tail” in the MTF, allowing one to resolve high-frequencies (albeit with reduced contrast). As opposed to the G channel, the R and B channels may not have such a peak, since the image is defocused at these wavelength ranges. However, if there is an overlap in the spectral responsivities of the RGB channels (as typically occurs in a Bayer filter, see also Figure S2), there may still be some sensitivity of the R and B channels at 550 nm (center of the G channel), giving rise to a sharp PSF peak there too. Of course, the peak will be lower, because of the reduced sensitivity.

### 5.3 Spatially multiplexed achromatic metalens

To demonstrate the performance of a spatially multiplexed achromatic metalens, we used the design presented in [47], based on a-Si nano-blocks, which was applied there to an achromatic hologram. Here we apply it to a metalens, with the same parameters as the second achromatic diffractive lens design of Section 8.2 of the Supplementary (EFL 1 mm, NA 0.18). The nanoantenna spectral response, shown in Figure S6, is narrower than the camera spectral response, shown in Figure S2, thus dominating the response. We therefore used the nanoantenna spectral response for the wavelength weights in Zemax.

**Figure 5:**
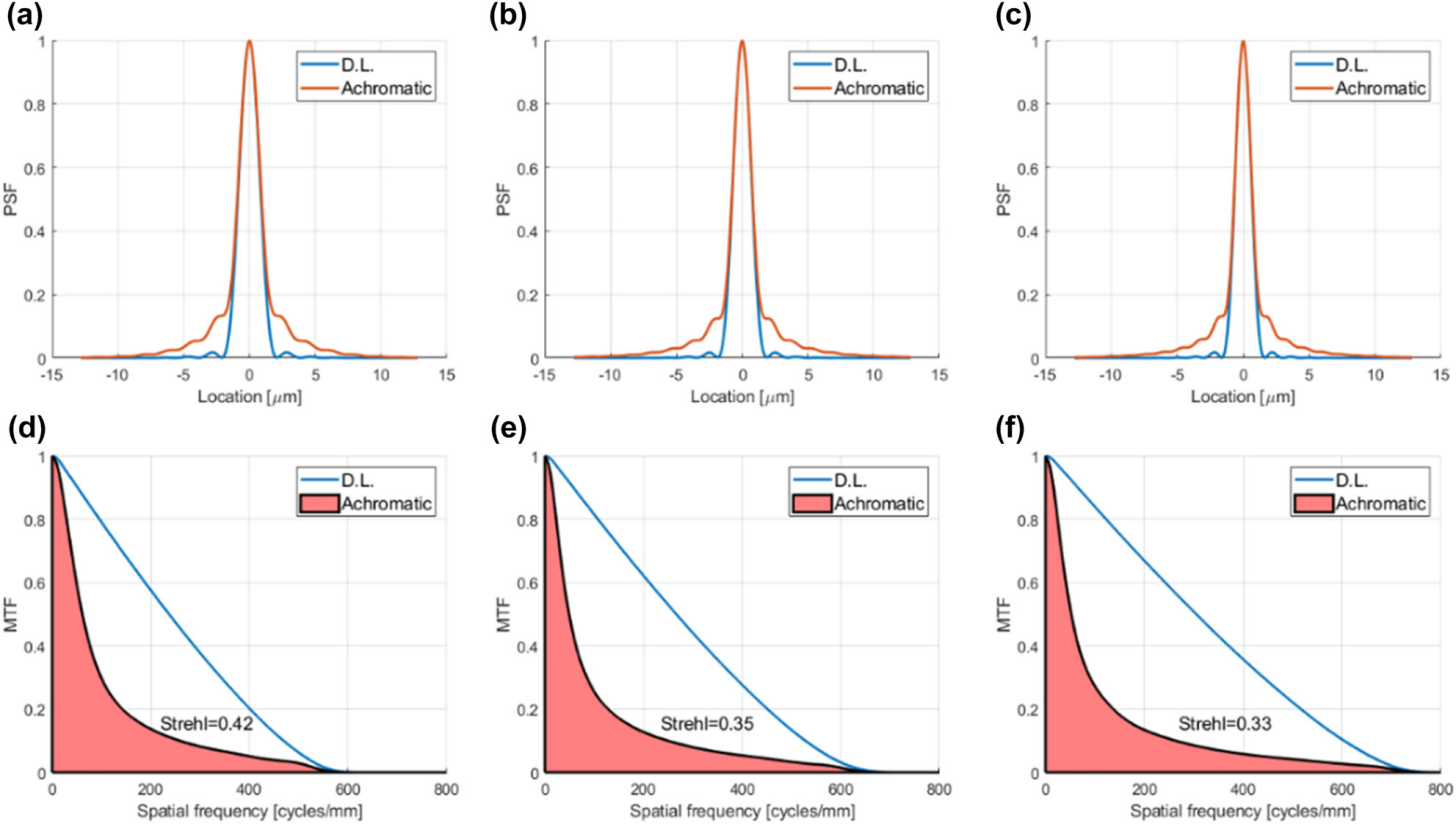
PSFs and MTFs of spatial multiplexed achromatic metalens over RGB spectral ranges, defined according to spectral response of nanoantennas given in [47], with lower and upper cutoffs at 450 nm and 650 nm, respectively. (a) R PSF cross section (b) G PSF cross section (c) B PSF cross section (d) R MTF (e) G MTF (f) B MTF.

In Figure 5 the MTFs for the spatial multiplexed achromatic metalens simulated in Zemax are shown. The simulation was performed by simulating a diffractive lens in Zemax and adjusting the phase function separately for each of the RGB channels, to obtain optimal focus at the common focal plane. In this manner we obtain similar performance for each of the RGB channels, with minor variations due to the differences in relative spectral width of the channels.

**Figure 6:**
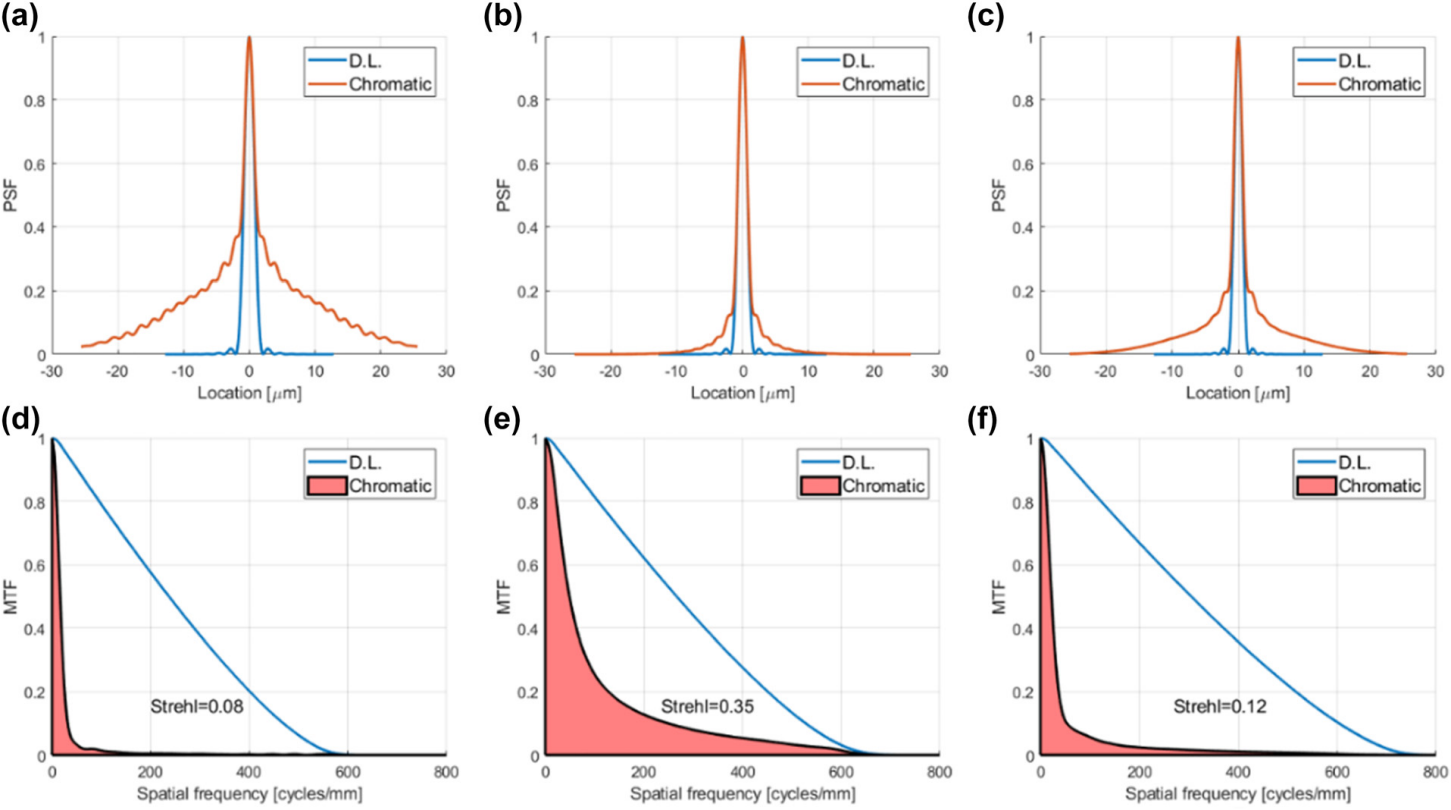
PSFs and MTFs of conventional diffractive lens over RGB spectral ranges, defined according to spectral response given in [47], with lower and upper cutoffs at 450 nm and 650 nm respectively (a) R PSF cross section (b) G PSF cross section (c) B PSF cross section (d) R MTF (e) G MTF (f) B MTF.

In Figure 6 the MTFs for an equivalent conventional diffractive lens are shown. As expected, the G channel MTF is identical to that of Figure 5. However, the B and R channels are now greatly defocused, giving rise to the low MTFs shown in Figure 6(d) and (f). Despite this, the overall performance (EOPMcolor) of the conventional diffractive lens, shown in Table 3, is better than the achromatic metalens. The reason for this is the low efficiency of the achromatic metalens, especially at the shorter wavelengths (B and G channels), because of the absorption in the silicon nano-blocks.

Even though we do not see improved performance here, the spatial multiplexing method may have potential if the efficiencies can be improved by using a more transparent material. This was done in [48] using GaN nanoantennas. However, since the polarization conversion efficiencies are high over the entire spectrum for all three antenna types (R, G, and B), it seems there is high crosstalk there. Another option is longitudinal cascading of metalenses, which was done in [11] using metallic nanoantennas. However, this design probably suffers from low efficiency. If done with dielectric nanoantennas, the challenge is to get each of the RGB layers to interact only with the relevant spectral range and be transparent to the other ranges.

**Table 3:** Comparison of achromatic metalens (AML) based on nanoantennas of [47] to equivalent conventional diffractive lens (CDL).

Design	1					
λmin [µm]	0.45					
λmax [µm]	0.65					
NA	0.18					
EFL [µm]	1000					
Dia. [µm]	336					
Fresnel no.	60.4					
Airy rad [µm]	1.86					
Chr. rad [µm]	33.3					
	**AML**			**CDL**		
	**R**	**G**	**B**	**R**	**G**	**B**
Efficiency	0.15	0.05	0.02	0.58	0.59	0.56
1D Strehl	0.42	0.35	0.33	0.08	0.35	0.12
OPM	0.16	0.08	0.04	0.06	0.27	0.09
EOPM	4.39	2.21	1.05	1.62	7.30	2.22
EOPMcolor	1.57			1.91		

### 5.4 Extended depth of focus metalens

**Figure 7:**
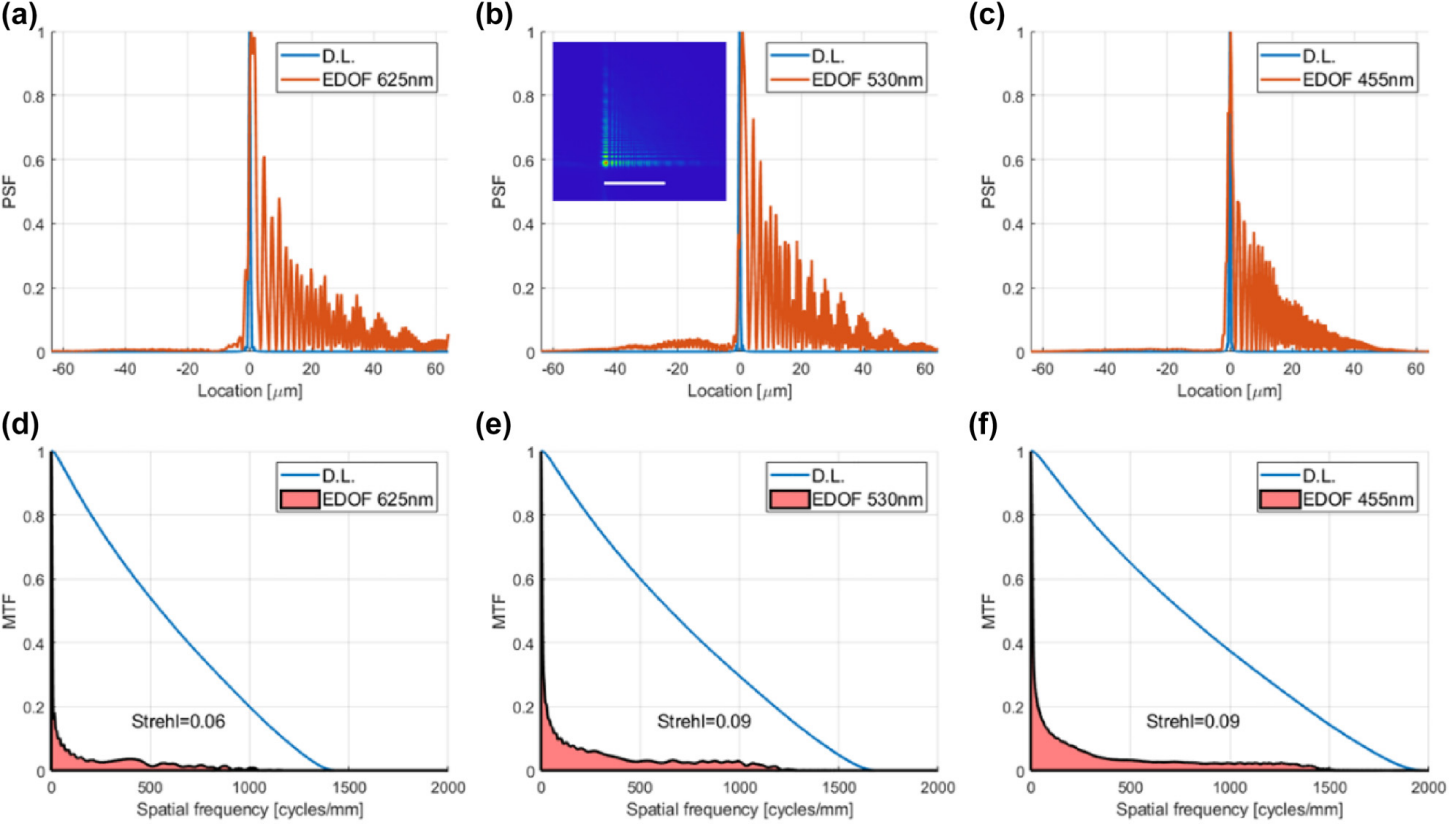
PSFs and MTFs of EDOF lens at wavelengths used in [9]. (a) 625 nm PSF cross section (b) 530 nm PSF cross section. Inset – 2D PSF. Scale bar is 30 µm (c) 455 nm PSF cross section (d) 625 nm MTF (e) 530 nm MTF (f) 455 nm MTF.

The idea behind this metalens is to add a cubic phase function to the basic focusing hyperbolic phase function, thus creating an extended depth-of-focus (EDOF). If the defocus resulting from chromatic aberration is smaller than the depth-of-focus, a focal plane where all wavelengths are in focus can be found. In [9] an EDOF metalens is presented for color imaging. Since the lens parameters are given in the paper, including the cubic phase coefficient, we were able to simulate the lens in Zemax. The monochromatic PSFs and MTFs obtained by our Zemax simulation for the three center-wavelengths used in [9] are shown in Figure 7. See Supplementary Section 8.4 for comparison to the results shown in [9].

**Figure 8:**
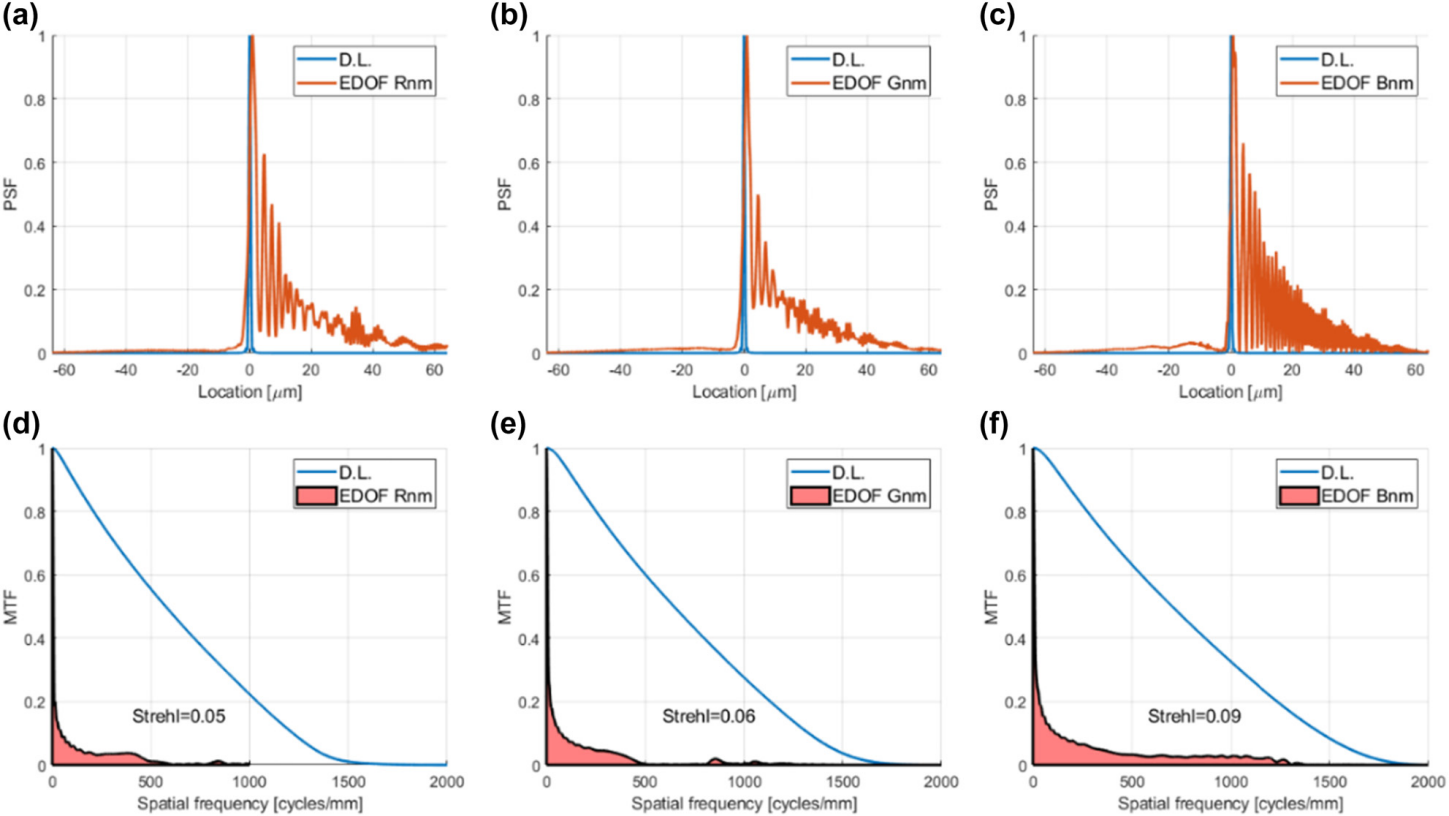
PSFs and MTFs of EDOF lens over RGB spectral ranges, defined according to spectral sensitivity of Thorlabs DCC1645C color camera. (a) R PSF cross section (b) G PSF cross section (c) B PSF cross section (d) R MTF (e) G MTF (f) B MTF.

In Figure 8 we show the simulated MTFs for the same lens, but over the RGB spectral ranges of the Thorlabs color camera. The MTFs for the R and G channels (Figure 8(d) and (e)) are worse than those of the monochromatic wavelengths shown in Figure 7(d) and (e). This seems to be a result of the fact that, as can be seen in Figure 1E of [9], the focal spot shifts in the transverse direction for different focal planes. This means that the same effect will occur at a single focal plane for different wavelengths. So, if we have polychromatic light, the overall MTF will be worse than the average MTF of the different wavelengths (The effect is accounted for by the overall OTF, which is the weighted average of the OTFs. The phase of the OTF accounts for the transverse shift of the PSFs). Therefore, this type of EDOF lens may not be a good method for creating an achromatic lens for imaging purposes.

**Figure 9:**
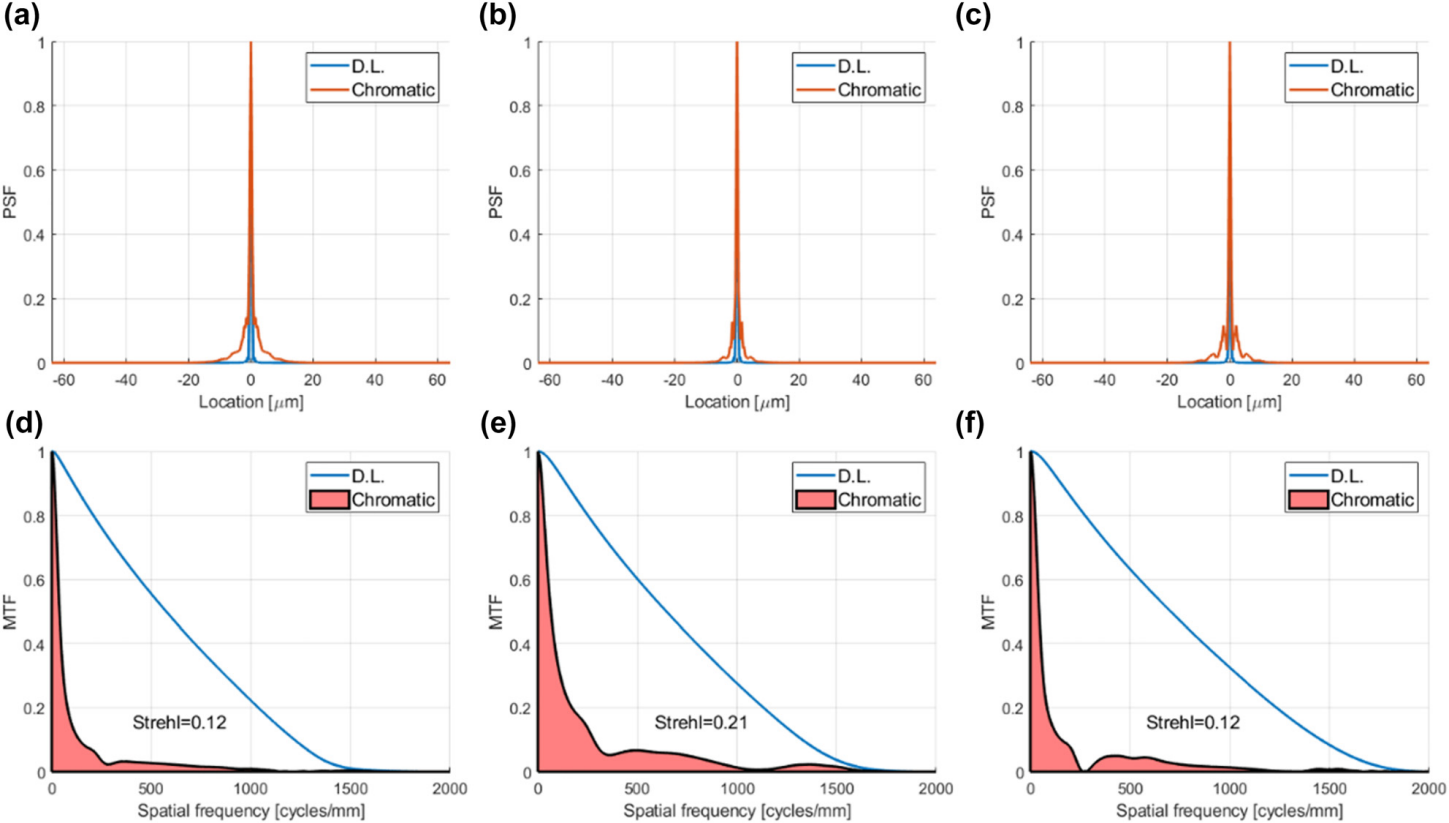
PSFs and MTFs of conventional diffractive lens (with parameters equivalent to EDOF lens) over RGB spectral ranges, defined according to spectral sensitivity of Thorlabs DCC1645C color camera. (a) R PSF (b) G PSF (c) B PSF (d) R MTF (e) G MTF (f) B MTF.

Last, but not least, in Figure 9 we show the MTFs for an equivalent conventional diffractive lens. Poor as the MTFs are, because of chromatic aberration, they are still better than the EDOF MTFs of Figure 8. As a result, the overall performance (EOPM and EOPMcolor) shown in Table 4 is better for the conventional diffractive lens. See Supplementary Section 8.4 for discussion of a symmetrical EDOF design published later by the same authors, and the performance metric they used.

**Table 4:** Comparison of achromatic metalens (AML) based on EDOF lens of [9] to equivalent conventional diffractive lens (CDL).

Design	1					
λmin [µm]	0.45					
λmax [µm]	0.65					
NA	0.45					
EFL [µm]	200					
Dia. [µm]	200					
Fresnel no.	85.7					
Airy rad [µm]	0.75					
Chr. rad [µm]	18.2					
	**AML**			**CDL**		
	**R**	**G**	**B**	**R**	**G**	**B**
Efficiency	0.85	0.85	0.85	0.58	0.59	0.56
1D Strehl	0.05	0.06	0.09	0.12	0.21	0.12
OPM	0.05	0.06	0.08	0.09	0.16	0.09
EOPM	1.97	2.63	4.32	3.9	7.6	4.7
EOPMcolor	2.27			4.27		

## 6 Conclusions

The main goal of this paper is to facilitate future progress in the field of achromatic flat lenses by introducing appropriate performance metrics and stressing the importance of comparing achromatic flat lenses to the benchmark equivalent conventional flat lens. A secondary objective is to assess the potential of the various types of achromatic flat lenses based on these metrics, as an aid to researchers in their attempt to channel their research in the best direction.

Remarkably, the single channel (not color) broadband achromatic metalenses we have analyzed in this paper have worse overall imaging performance than the equivalent conventional diffractive lens. As for the achromatic diffractive lenses we analyzed, it is difficult to determine their true performance, because a proper evaluation of their resolution was not made (only FWHM criterion was used). However, we suspect that given a proper evaluation, they would still not show an improvement compared to their conventional diffractive lens counterparts [17]. We note that in all analyzed cases, the overall transmission and veiling glare were not measured, and we assumed that the only transmitted light is that of the design order, so the calculated achromatic flat lens OPMs are optimistic.

For color imaging, achromatic flat lenses seem to have greater potential for improvement over conventional flat lenses. This is because in color imaging, the resolution of the R and B channels is greatly reduced for a conventional flat lens, precluding attainment of high color fidelity, while with achromatic flat lenses the R and B channels can have resolution like the G channel, allowing high color fidelity. Still, for the achromatic flat lenses analyzed, the overall performance did not exceed that of a conventional flat lens.

What is currently limiting the performance of spatially multiplexed achromatic metalens designs (an example of which was analyzed in Section 5.3) is low efficiency because of material absorption in silicon. The ideal solution would be a high-index material that is transparent in the visible range, which can allow high efficiency and low coupling between channels to be obtained. The problem is that there is no such material with refractive index as high as silicon. Promising candidates such as TiO_2_ and GaN have not yet been applied to spatially multiplexed achromatic metalenses (GaN has been applied to a color-routing metalens, but there seems to be an issue of crosstalk between channels [48]).

Another important issue with flat lens performance, which we did not explore in this paper (although we did propose a metric for it in Section 4), is the ability to retain the performance over a significant FOV. This has been addressed by using a removed aperture stop [49–51] and more recently using a phase induced effective aperture [52]. Regarding the latter method, we believe that an analysis based on the method presented in this work will show its limited usefulness, since despite the small FWHM, the Strehl ratio is very low, because of large spherical aberration.

We look forward to seeing new applications and improved performance in the field of flat lenses. We believe that progress in this field, enabling a transfer of achromatic flat lens technology to industry, necessitates not only innovative physics, but also down-to-earth good engineering practice. There are many advanced designs that can be achieved, and several additional degrees of freedom that can be utilized. We hope to see such implementations soon.

## Supplementary Material

Supplementary Material Details
