# Supplementary material for: Generalized metric for broadband flat lens performance comparison
Source: Nanophotonics. 2022 Jul 14;11(16):3559–74. doi: 10.1515/nanoph-2022-0196 (PMC11502021; doi:10.1515/nanoph-2022-0196)
Supplement: Supplementary file 1 — Supplementary Material Details [file j_nanoph-2022-0196_suppl.docx]

Supplementary information for:

Generalized metric for broadband flat lens performance comparison

# Diffraction efficiency vs. focusing efficiency

In this section we explain how the diffraction efficiency *η* should be simulated and measured to obtain correct results, which can be used in our merit function. The concept of diffraction efficiency is well known and for the case of a lens is defined as the fraction of the incident energy (within the lens aperture) that goes to the design diffraction order [1–3], usually the first order. However, in recent years a new performance metric known as “focusing efficiency” has come into use in the flat lens community. To the best of our knowledge, the first to introduce this metric was Arbabi et al. in [4]. The focusing efficiency is defined there as the fraction of the incident energy that reaches the image plane region within a radius of 3×FWHM, centered on the center-of-mass of the focal spot.

To our understanding the intention of the authors of [4] was to define a measurable quantity that will closely represent the diffraction efficiency. Apparently, they assumed their lens produces near-diffraction-limited resolution, i.e., the focal plane intensity distribution of the first diffraction order is close to an Airy pattern. For an Airy pattern spot, 93.7% of the energy is contained within a radius of 3×FWHM, so this is a reasonable approximation of the diffraction efficiency. In other publications different radii were used in the definition of focusing efficiency, as indicated in [5].

The radius used for the focusing efficiency calculation is usually given in terms of the FWHM (e.g., 3×FWHM), and not of the Airy radius, despite their being quite similar for a diffraction limited lens. We conjecture the reason for this is that since the FWHM is related to the actual size of the central disk of the focal spot, it was thought that an area with a radius several times larger than the diameter of this disk will likely encompass almost all the energy, even in the case of a lens whose resolution is far from the diffraction limit. However, this is not usually true. As long as the aberrations are small enough that the Fraunhofer approximation holds [6], the FWHM represents the diameter of the central disk, and is fairly constant. However, these aberrations cause more energy to be directed to distant sidelobes, which may still fall outside the measured radius. If the aberration is larger than the Fraunhofer limit, there may be no central disk, so the FWHM is quite arbitrary, and 3×FWHM certainly cannot be assumed to contain almost all the energy.

To summarize, focusing efficiency, when defined as the fraction of incident energy contained within a radius of several FWHM at the image plane, can give a reasonable estimate of the diffraction efficiency if the lens has near diffraction limited resolution. However, if the lens is not nearly diffraction limited, it will give a result which represents a mixture of two effects: (a) Wavefront distortion of the design order, causing resolution degradation. (b) Diffraction efficiency, causing loss of signal and veiling glare, which results in SNR degradation. The weight of the contribution of each of the effects will depend on the radius of the chosen image plane region. Unless this type of measurement exactly matches the application (such as coupling to a multimode fiber with a core radius equal to the radius of the measurement region) such a measure of performance is not useful. Therefore, if one wants to measure true efficiency, a different method should be found for defining the region in the image plane over which the energy is collected. How should this region be defined?

The answer to this question is quite simple. The signal should be collected from the region over which the PSF is still varying, until it has “flattened out”. For example, for the CDL PSF of Fig. 3(a) of the main text, this happens at about 10µm from the center. In Fig. 3(b) it happens at about 20µm, and in Fig. 3(c) it does not happen within the range shown in the graph. Note that this measurement/simulation region does not necessarily correspond to 3×FWHM, 10×FWHM, etc., since it depends on the shape of the PSF.

The PSF region defined to be used in efficiency measurement is the **same region** that must be used for simulation/calculation of the MTF based on the Fourier transform of the PSF (see following section of this Supplementary for definition of MTF and PSF. As a note, the MTF of Fig. 3(f) was calculated based on a larger region than that shown in the PSF graph of Fig. 3(c)). Since almost all reports of flat lenses present PSF/MTF results, the size of this region must be known. All that now remains is to measure the efficiency over this same region.

There are certain types of flat lenses where it does make sense to talk about focusing efficiency, e.g., multi-order diffractive (MOD) lenses [7–9] and inverse design multi-level diffractive lenses (MDLs) [10,11]. In these types of lenses, there is no single design order, but rather a few or many orders that operate at varying levels of efficiency for different wavelengths within the design spectral range. Therefore, the classic definition of diffraction efficiency is not suitable, and it is more appropriate to use the term ‘focusing efficiency’. However, when it comes to simulating and measuring the focusing efficiency, the above guidelines are still relevant.

When evaluating the efficiency, it is also imperative to compare the “signal” to the **overall incident light**, and not only to a small part of it that happens to fit into the camera’s active area or into the simulation window [12]. A convenient way of performing an efficiency measurement using the same setup used to measure PSF/MTF, without the need for a pinhole, is described in [13].

# Resolution metric – Strehl ratio vs. FWHM

It has long been recognized by the ultra-short laser pulse community that although the FWHM historically became popular as a measure of pulse temporal width, is not a good choice for most cases, since the pulse may have temporal substructure or broad wings causing a considerable part of the energy to lie outside the 50% intensity range. The metrics of choice are, therefore, the standard deviation of the pulse (when regarded as a probability density function) or the width of some type of autocorrelation function [14]. The equivalent problem occurs in the realm of spatial resolution, where FWHM seems to be a popular metric, although it is a very poor measure of resolution.

Examples of the problem with FWHM in the realm of spatial resolution are demonstrated in Fig. S1, for aberrated focal spots resulting from chromatic and spherical aberration of a conventional diffractive lens (CDL). The FWHM is compared to the widely accepted resolution metric of modulation-transfer-function (MTF), which is a function of spatial frequency [15], and to the Strehl ratio, which is a single number measure of how close the performance is to the diffraction limit. As can be seen in the figure, the FWHM fails to give any indication of the tremendous drop in resolution as a result of these aberrations (in fact, in our example the FWHM improves as a result of the spherical aberration, by an effect similar to that found in super-oscillation lenses [16]).


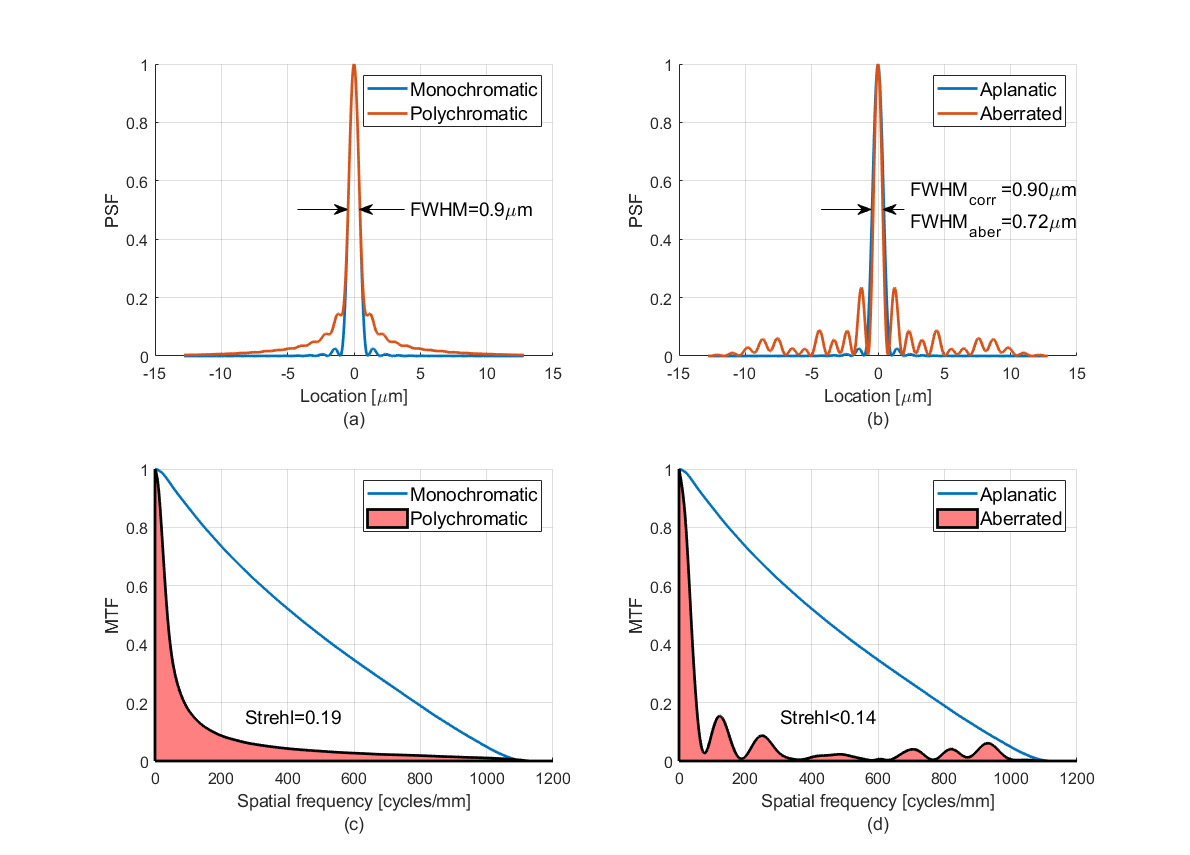


Figure S1: FWHM resolution metric compared to MTF and Strehl ratio. The comparison is performed on a CDL of 3.36mm focal length, F/1 (NA 0.45), operating at wavelengths around 800nm. (a) PSF cross sections for monochromatic (diffraction limited) case and 20nm bandwidth (top-hat shaped spectrum) case. (b) PSF cross sections for monochromatic diffraction limited operation and the for the same lens with the two highest phase coefficients removed – thus introducing spherical aberration. (c) MTFs for PSFs of ‘a’. (d) MTFs for PSFs of ‘b’. The Strehl coefficients shown are the 1D version, equal to the pink shaded area under the MTF graph for the chromatic aberration case, and smaller than it for the spherical aberration case, since the OTF is negative for some frequencies.

MTF, on the other hand, is the industry-standard spatial resolution metric for imaging applications. The MTF is the modulus of the optical-transfer-function (OTF), which in turn is the Fourier transform of the point-spread-function (PSF), the spatial intensity profile of the focal spot. The MTF gives the signal attenuation at each spatial frequency. It is analogous to the spectral amplitude of a laser pulse in the temporal realm.

Interestingly, the spectral intensity or amplitude is considered a poor indicator of temporal pulse width, since it lacks the phase information, which can cause significant pulse broadening. However, in the spatial realm, the MTF is considered a good spatial resolution metric, even though the phase information (given by the phase transfer function – PTF) is lost by the modulus operation. It seems that the reason for this is that while in temporal pulses it is quite common to have dispersion that affects the phase but not the amplitude of the spectral components, common lens aberrations will affect the amplitude as well as the phase of the OTF. Therefore, while the phase component of the OTF can have significant effect on image quality [15], a situation where the MTF is high, but the PSF is broad because of the PTF, is not realistic. Note that as opposed to the temporal realm, where the phase is difficult to measure, it is quite easy to obtain the PTF since it is simply the angular component of the complex OTF. Still, this information is usually ignored.

The Strehl ratio is defined as the ratio of the PSF peak to the diffraction limited PSF peak (when the PSFs are normalized so that the area under the PSF graphs is equal to 1). Because of the Fourier relation between MTF and PSF, the Strehl ratio is equal to the volume under the 2D MTF graph, relative to the volume under the diffraction limited 2D MTF (for real and positive OTF). The Strehl ratio is an optimal measure of performance for many non-imaging applications and certain imaging applications, such as astronomy or microscopy, where one needs to detect point-objects above the noise level. However, it is not ideal for typical wide-scene imaging applications. This is because the Strehl ratio is related to the volume under the 2D MTF, so high spatial frequencies are unduly given higher weight than the low spatial frequencies. Therefore, for most imaging applications it is more appropriate to use the one-dimensional (1D) version of the Strehl ratio, known also as the Struve ratio [17], which is defined as the ratio of the line-spread function (LSF) peak to the diffraction limited LSF peak (the LSF is the one-dimensional version of the PSF obtained by summing the 2D PSF in one direction). As a result of the Fourier relation between the LSF and the 1D MTF, the LSF peak is equal to the area under the 1D MTF graph, for real and positive OTF [18,19]. All the examples shown in this paper are on-axis, therefore they have a symmetrical LSF, and therefore a real OTF. If there are no zeros in the OTF up to the frequency cutoff, the OTF is also positive. This is the case for all the examples in the paper, except that shown in Fig. S1(d), therefore we indicated there that the Strehl ratio is smaller than the area under the MTF and not equal to it.

Note that even under the condition of real and positive OTF, the relation between LSF peak and MTF is valid only if the peak of the LSF/PSF occurs on-axis. For an on-axis object, this will be the case if the aberrations are small enough for the Fraunhofer approximation to be accurate. If this is not the case, it is better to define the 1D Strehl ratio as the ratio of the areas under the MTFs, which is the convention we followed in this paper (this can be useful also for the off-axis case, where usually the PSF/LSF is not symmetrical, so again the peak may be off-axis). Usually, the LSF and MTF are calculated in horizontal and vertical directions, so we have horizontal and vertical 1D Strehl ratio. However, in this paper we deal only with radially symmetric systems, so there are single MTF and Strehl ratio values.

To summarize this section, we are not saying that the FWHM metric should never be used. It may be suitable for certain applications, such as evaluation of super-oscillation lenses for super-resolution imaging. However, this is the exception rather than the rule. For a conventional imaging lens, the FWHM is a very insensitive parameter, related primarily to the system central wavelength and relative aperture, and having little relation to the optical performance. The preferred single number performance parameter is the 1D or 2D Strehl ratio, depending on the application.

# Zero frequency contrast measurement

As mentioned in section 2 of the main text, background light from spurious diffraction orders not only does not contribute to the signal but adds noise. Therefore, evaluating the level of background light is an important part of flat lens performance evaluation. The light from spurious diffraction orders is highly defocused, and therefore exists in the area beyond where the PSF flattens out. This light contributes to overall contrast reduction, i.e., it multiplies the entire MTF by a constant factor, which can be approximated by the $\eta/T$ factor mentioned in section 2 of the main paper [2]. This factor is equal to the contrast of very low spatial frequencies (since it is equal to the value of the un-normalized MTF at zero frequency). Note that we use the terms contrast and modulation interchangeably here, both defined by Eq. S2.

How can this factor be measured? It cannot be measured as part of the MTF, because the field-of-view (FOV) of the PSF measurement setup is too small (to obtain adequate spatial sampling of the PSF, large magnification is used, thus the FOV at the image plane is small). Therefore, the common engineering practice is to measure PSF/MTF by looking at the region until where the PSF flattens out, and to normalize the MTF so that at the zero frequency it is equal to 1. The zero-frequency contrast is relegated to a sperate measurement [12].

An indirect method of measuring the zero-frequency contrast, based on the $\eta/T$ approximation, is by placing a large detector immediately following the lens, to collect all the transmitted light. The ratio of this signal to that obtained from the incident light (using the same lens aperture) will give *T*, while *η* is obtained from the previously discussed efficiency measurement (see section 1 of this Supplementary).

The direct method of measuring low spatial frequency contrast is known as veiling glare (VG) measurement. This measurement is performed with the lens coupled directly to a camera located at the image plane. A target composed of a large dark area on white background is viewed by the system. The veiling glare is then defined as [20]:

| $VG\equiv\frac{black level}{white level}$ | (S1) |
| --- | --- |

Where the black and white level are defined relative to the ‘capped black’ level of the camera, which is the average signal level output by the camera when no light is incident.

The modulation of a signal is defined as:

| $M\equiv\frac{white level-black level}{white level+black level}$ | (S2) |
| --- | --- |

By combining Eq. S1 and S2 we obtain Eq. S3, which gives us the low frequency modulation, as a function of the measured VG (one can of course just use Eq. S2 directly, and skip Eq. S1 and S3).

| $M\equiv\frac{1-VG}{1+VG}$ | (S3) |
| --- | --- |

As explained, when the MTF is measured or calculated, it is generally normalized so that at zero frequency it is equal to 1. The parameter *M* represents the absolute (un-normalized) MTF value at zero frequency, via Eq. S2 or S3. Therefore, we can multiply the MTF by this factor, to obtain the absolute MTF. Despite this possibility, it is customary to separate the two effects, i.e., use the normalized MTF and the VG as two separate metrics of system performance, the first representing the system ‘resolution’, and the second representing the system low frequency ‘contrast’. Note that for our metric we need both *η* and *T,* so it is not enough to measure only VG. We must measure at least 2 out of these three parameters, preferably *η* and VG (from the VG we can obtain *M*, and from *M* and *η* we can obtain the “true” effective *T* that contributes to the background signal).

The tricky part about VG measurement is that the level of background radiation depends on the ambient illumination. Therefore, one must attempt to match the measurement setup to the application, and of course perform functional tests as well. However, in the case of flat lenses the VG is usually stemming from spurious diffraction orders, and not from parasitic reflections from mechanical housing and optical surfaces. Therefore, it should be less sensitive to ambient illumination conditions.

# Derivation of color EOPM

As explained in the main paper, to obtain good color fidelity we need to have good SNR and resolution, represented by the EOPM, for each one of the RGB channels. This means that in addition to requiring high average EOPM for the three channels, we need to require low standard deviation among them.

The average EOPM is given by:

| $avg\left( EOPM \right)=\frac{{EOPM}_{R}+{EOPM}_{G}+{EOPM}_{B}}{3}$ | (S4) |
| --- | --- |

The standard deviation is given by:

| $std\left( EOPM \right)=\sqrt{\frac{\left( {EOPM}_{R}-avg \right)^{2}+\left( {EOPM}_{G}-avg \right)^{2}+\left( {EOPM}_{B}-avg \right)^{2}}{2}}$ | (S5) |
| --- | --- |

For a case where we have non-zero EOPM in only one channel, say the green channel:

| ${EOPM}_{R}={EOPM}_{B}=0\overset{\Rightarrow}{} avg\left( EOPM \right)=\frac{{EOPM}_{G}}{3}$  $\overset{\Rightarrow}{}{EOPM}_{G}=3avg\left( EOPM \right)$ | (S6) |
| --- | --- |

Substituting Eq. S6 into Eq. S5 we obtain:

| $std\left( EOPM \right)=\sqrt{3} avg\left( EOPM \right)$ | (S7) |
| --- | --- |

Therefore, we choose the color merit function to be the average EOPM, multiplied by the correction factor 1-$std\left( EOPM \right)/\sqrt{3} avg\left( EOPM \right)$. This factor is equal to 1 when the standard deviation is zero, i.e., maximum color fidelity, and 0 when the standard deviation is as calculated above for the case of signal from only one channel.

# Derivation of extended FOV EOPM

The information content of an image, in bits per unit area, is given by [21]:

| $C=\iint_{-\infty}^{\infty} log\left[ 1+\frac{{MTF}^{2}(\nu_{x}{,\nu}_{y})\cdot P(\nu_{x}{,\nu}_{y})}{N(\nu_{x}{,\nu}_{y})} \right]d\nu_{x}d\nu_{y}=\iint_{-\infty}^{\infty} log\left[ 1+{MTF}^{2}(\nu_{x}{,\nu}_{y})\cdot{SNR}^{2}(\nu_{x}{,\nu}_{y}) \right]d\nu_{x}d\nu_{y}$ | (S8) |
| --- | --- |

Where *P* is the signal power spectral density, and *N* is the noise power spectral density. Note the similarity between Eq. S8 and Eq. 1, in that we have the product of the MTF and SNR. The main difference is the *log* function that is applied before the integration is carried out. It is tempting to neglect the 1 inside the *log*, since usually the SNR will be much larger than 1. However, this cannot be done, since at higher spatial frequencies the MTF will go down towards zero. The 1 inside the *log* is necessary for these high spatial frequencies, since otherwise the *log* will take on negative values, that will incorrectly reduce the amount of information, instead of only not adding to it. We assume “white” signal and noise within the system bandwidth, so the SNR is constant, giving us Eq. S9:

| $C=\iint_{-\nu_{co}}^{\nu_{co}} log\left[ 1+{SNR}^{2}\cdot{MTF}^{2}(\nu_{x},\nu_{y}) \right]d\nu_{x}d\nu_{y}$ | (S9) |
| --- | --- |

Where $\nu_{co}={2NA}/\lambda$ is the diffraction limit cutoff frequency of the lens. Assuming a shot noise limited system, the SNR can be calculated according to [22]:

| $SNR=\left[ \frac{A_{pix}t}{hc}\int\pi QE(\lambda)\cdot\lambda\cdot L_{\lambda}(\lambda)\times\eta(\lambda)\times{(NA)}^{2}d\lambda\right]^{1/2}$ | (S10) |
| --- | --- |

Where *A_pix_* is the pixel area, *t* is the camera integration time, *h* is Planck’s constant, *c* the speed of light in vacuum, *QE* the camera quantum efficiency, *L_λ_* the object spectral radiance, *η* the flat lens efficiency, and *NA* is the numerical aperture of the lens. To simplify, we assume the spectral range is small enough so that the various parameters in the integral can be considered constant. We then obtain:

| $SNR=NA\left[ \frac{A_{pix}t}{hc}\pi QE\cdot L_{\lambda}\cdot\eta\cdot\lambda\cdot\Delta\lambda\right]^{1/2}$ | (S11) |
| --- | --- |

The above derivation assumes no background illumination that contributes to noise but not to signal. To include the background noise, we must replace *η^1/2^* in Eq. S11 with $\eta/\sqrt{T}$. When using this merit function, we must compare based on absolute differences, not relative, since the *log* function converts products into sums. To obtain the overall information we multiply the information per unit area, *C*, by the image area *A*:

| $EOPMfov=A\cdot C$ | (S12) |
| --- | --- |
|  |  |

The drawback of this merit function is that it is not possible to separate the lens from the camera and the scene illumination. So, to compare between types of flat lenses, we must consider the specific application. Regarding the image area, we can compare lenses over equal image areas, without loss of generality. Since typically the MTF will change over different image regions, we suggest breaking up the image into several areas (rings in the case of radially symmetric systems) and summing the merit functions for the different areas. If the camera has not yet been determined, one can make reasonable assumptions about it for use in the calculation (such as *QE*=1, *t* based on the desired frame rate, reasonable pixel size based on the state of the art, etc.). However, one must have an estimate of the ambient lighting since it too will affect the choice of lens aperture.

# Commercial optical design software simulation

In the simulations shown in this paper, the MTF and Strehl ratios are polychromatic, i.e., calculated over a certain spectral range. The optical design software performs polychromatic simulations by simulating many discrete wavelengths, defined by the user, and then performing a weighted average, according to weights defined by the user. We made sure to use enough wavelengths to simulate a continuous spectrum.

All the MTF simulations shown in the paper are for a diffractive surface. The radially symmetric ‘Binary2’ surface type was used in Zemax for all the simulations. For the extended depth of focus (EDOF) metalens (section ‎8.4 of this Supplementary), a non-radially symmetric ‘Binary1’ surface was added, to describe the cubic phase.

All the PSFs and MTFs in this paper were calculated using the Huygens PSF and MTF option in Zemax. This was necessary since the aberrations are large in many of the cases, to the level where the Fraunhofer approximation is no longer valid in the image plane [6].

For high-NA lenses the computation of the PSF/MTF is more complicated since the Z-component of the electric-field must be considered [23–25]. In addition, in the case of a flat lens we obtain higher angular ray density, and therefore higher intensity of light, at the edges of the aperture. This creates a pupil apodization effect, like that of a central obscuration [26]. While Zemax can account for these effects, we opted to use Code V for the high-NA cases, since we found a difference between the two software packages, and our impression is that Zemax is overemphasizing the pupil apodization (this subject is still under investigation). Therefore, the diffraction limited MTFs of Fig. 4(f) and 5(f) do not have the typical shape of low NA diffraction limited MTFs. We used input circular polarization in Code V to simulate unpolarized light.

# CDL efficiency calculation

An analytic approximation of the first order diffraction efficiency for an *N* level diffraction grating, at the design wavelength and normal incidence, is given by [27] :

| $\eta=\frac{4n}{\left( n+1 \right)^{2}}{sinc}^{2}\left( 1/N \right) \left( 1-2\frac{N-1}{N}\frac{1}{n-1}\frac{\lambda}{d}tan\left( \theta\right) \right)$ | (S13) |
| --- | --- |

Where *n* is the substrate refractive index (which we took as 1.5 for our calculations), λ is the wavelength of light, *d* is the grating period, *θ* is the diffraction angle (assuming normal incidence), and $sinc\left( x \right)= \frac{sin\left( \pi x \right)}{\pi x}$. The first factor (before the *sinc*) accounts for Fresnel reflection from the diffractive surface, the *sinc* accounts for the effect of the phase sampling, and the last factor (in parentheses) accounts for the shadowing effect. This approximation is limited to *λ/d* that is not too small - for first order of diffraction it should be larger than about 2. For smaller values of *λ/d* (which cause the approximate result to be negative of complex), we took the pessimistic estimate of zero efficiency.

For the case of a diffractive lens, the local period changes along the radial direction of the aperture. Assuming a certain minimum feature size *Δ*, we can calculate the local number of phase levels, *N*, and therefore the local shadowing (the local period and diffraction angle are calculated using the grating equation). Integration over the aperture is then carried out to obtain the average efficiency over the full aperture. For the cases where the CDL is compared to an ADL, we used the same feature size for the CDL as was used in the published ADL (1µm, 4µm, and 0.35µm for designs 1, 2, and 3 of Table 2 respectively, and 3µm and 1.2µm for designs 1 and 2 of Table S2 respectively). For the cases where the CDL is compared to an AML, we used a feature size of 1µm.

In addition to the above efficiency calculation, we must also account for the efficiency degradation because of wavelength detuning. The first order diffraction efficiency averaged over a spectral range of Δλ, with uniform weighting, centered on the design wavelength λ_0_ is given by [28]:

| $avg(\eta)\approx1-\left( \frac{\pi\Delta\lambda}{6\lambda_{0}} \right)^{2}$ | (S14) |
| --- | --- |

The product of these two expressions was used to estimate the efficiency of the CDL designs presented in the paper in Tables 1 and 2, with *Δλ*, *λ_0_* per design (with design wavelength *λ_0_*=( *λ_min_+ λ_max_)/2*, for each case analyzed). The maximum number of phase levels is taken as 8, and the number is reduced to equal the integer part of (*d/Δ)* if it is less than 8. The phase levels are uniformly distributed between 0 and 2π, with jumps of 2π/*N*.

For the color imaging examples presented in section ‎8 of this supplementary, the wavelength detuing efficiency was cacluated using the formula for diffraction efficiency as a function of wavelength, since Eq. S14 is limited to uniform spectral weight (for the first order of diffraction, neglecting the material dispersion) [29]:

| $\eta={sinc}^{2}\left( {\lambda_{0}}/{\lambda-1} \right)$ | (S15) |
| --- | --- |

Using this formula, a weighted average, based on the spectral responsivity of the system, was performed. Note that strictly speaking, it is not correct to multiply the efficiency formulas based on number of levels/shadowing (Eq. S13) and based on wavelength (Eq. S14 and S15) to obtain the overall efficiency, since each assumes the other does not exist (level formula assumes single wavelength, and spectral formula assumes perfect blaze), when in fact there is coupling between the two effects. However, when a large enough number of levels is used, the coupling is negligible. A formula that gives the overall efficiency, including multi-level and wavelength effects is given in Eq. 3.1 of [28], but it does not include the shadow effect. The shadow effect could be added to that formula and integrated over wavelength to obtain more accurate results. However, for our purposes these approximations are sufficient.

Another point to mention is that low efficiency near the aperture edges will also affect the MTF, since it will act as aperture apodization. This would cause the diffraction limited MTF to drop, and the aberration blur to decrease, so the simulated MTF would be even closer to the diffraction limit (higher Strehl ratio), leaving less room for improvement because of chromatic correction. In our analysis we took the stringent assumption that the MTFs are not affected by the efficiency.

# Achromatic flat lenses for color imaging

## Dispersion engineered metalens

Here we analyze the performance of the AML presented in [30], based on dispersion engineered nanostructures, and compare to the performance of an equivalent CDL. This metalens has first-order parameters similar to Design 1 shown in Table 2 (originally published in [31]), but is improved in the sense that it is not polarization sensitive, and the spectral range is slightly extended. While in Table 2 we looked at performance over the entire spectral range, here we break up the spectral range into R, G and B channels, and look at the color imaging performance.

In Fig. S2 we show the spectral responsivity of the Thorlabs DCC1645C color camera, based on manufacturer data^[[1]](#footnote-1)^. We used this RGB spectral responsivity in our analysis but assumed an added long-pass filter with cut-on at 450nm was added in front of the lens (grayed out area in Fig. S2), since some of the designs analyzed were not optimized for shorter wavelengths. In Fig. S3 the equivalent CDL performance, over the RGB spectral bands of the camera, at a single common focal plane, is shown.


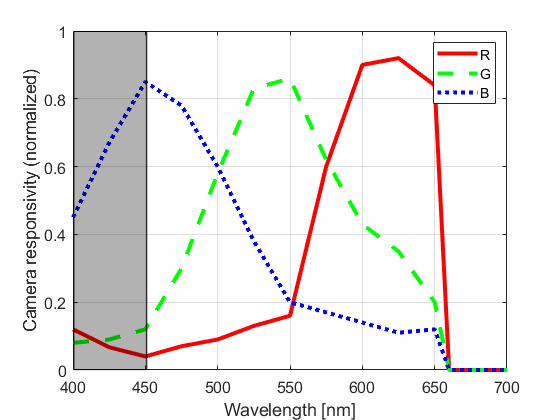


Figure S2: Spectral sensitivity of RGB channels of Thorlabs DCC1645C color camera. There is significant spectral overlap between the channels.


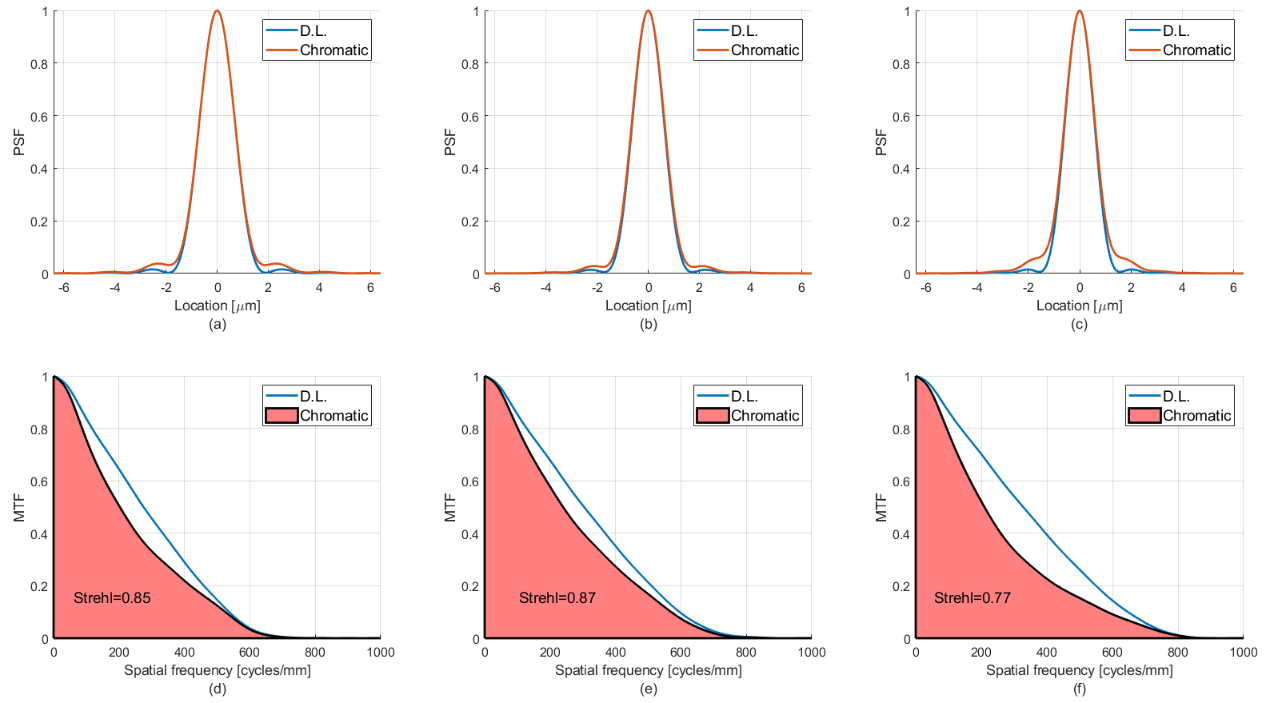
Figure S3: PSFs and MTFs of conventional diffractive lens, with EFL 67µm and NA 0.2, over RGB spectral ranges defined according to spectral sensitivity of Thorlabs DCC1645C color camera. (a) R PSF cross section (b) G PSF cross section (c) B PSF cross section (d) R MTF (e) G MTF (f) B MTF

Table S1. Comparison of AML of  [30] to equivalent CDL

| Design | 1 | | | | | |
| --- | --- | --- | --- | --- | --- | --- |
| λmin [µm] | 0.45 | | | | | |
| λmax [µm] | 0.65 | | | | | |
| NA | 0.2 | | | | | |
| EFL [µm] | 67 | | | | | |
| Dia. [µm] | 27 | | | | | |
| Fresnel no. | 5 | | | | | |
| Airy rad [µm] | 1.7 | | | | | |
| Chr. rad [µm] | 2.5 | | | | | |
|  | **AML** [30] | | | **CDL** | | |
|  | R | G | B | R | G | B |
| Efficiency | 0.3 | 0.3 | 0.3 | 0.61 | 0.62 | 0.59 |
| 2D Strehl | 0.8 | 0.8 | 0.8 | 0.65 | 0.83 | 0.67 |
| 1D Strehl | 0.9 | 0.9 | 0.9 | 0.85 | 0.87 | 0.77 |
| OPM | 0.49 | 0.49 | 0.49 | 0.66 | 0.68 | 0.59 |
| EOPM | 1.50 | 1.37 | 1.24 | 1.67 | 1.90 | 1.80 |
| EOPMcolor | 1.29 | | | 1.72 | | |

It can be seen from Table S1 that despite the improved resolution provided by the AML, the overall performance (OPM, EOPM and EOPMcolor) of the CDL is better. This is because, for the first order design parameters of the AML, the CDL does not have severe chromatic aberration (because of the low Fresnel number). Therefore, the small reduction in resolution is more than compensated by the improved efficiency of the CDL.

## Achromatic diffractive lens (ADL)

Here we analyze the performance of ADLs presented in [10], and compare to the performance of equivalent CDLs. We will look at two designs, one with NA of 0.05, and the other with NA of 0.18. In Fig. S4 we show the equivalent CDL performance for the first design (NA 0.05), over the RGB spectral bands of the Thorlabs DCC1645C color camera. In Fig. S5 we show the same for the second (NA 0.18) design. The performance comparison is summarized in Table S2.


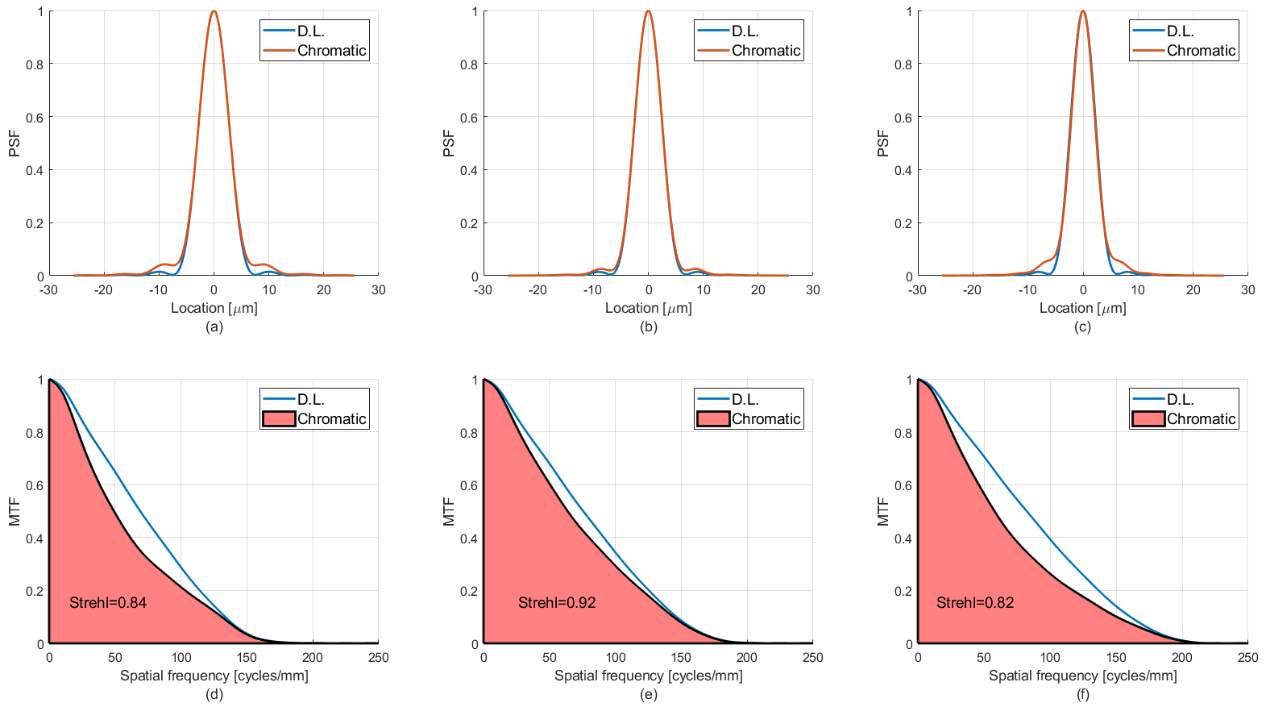
Figure S4: PSFs and MTFs of CDL equivalent to design 1 (NA 0.05) of  [10] over RGB spectral ranges, defined according to spectral sensitivity of Thorlabs DCC1645C color camera. (a) R PSF cross section (b) G PSF cross section (c) B PSF cross section (d) R MTF (e) G MTF (f) B MTF


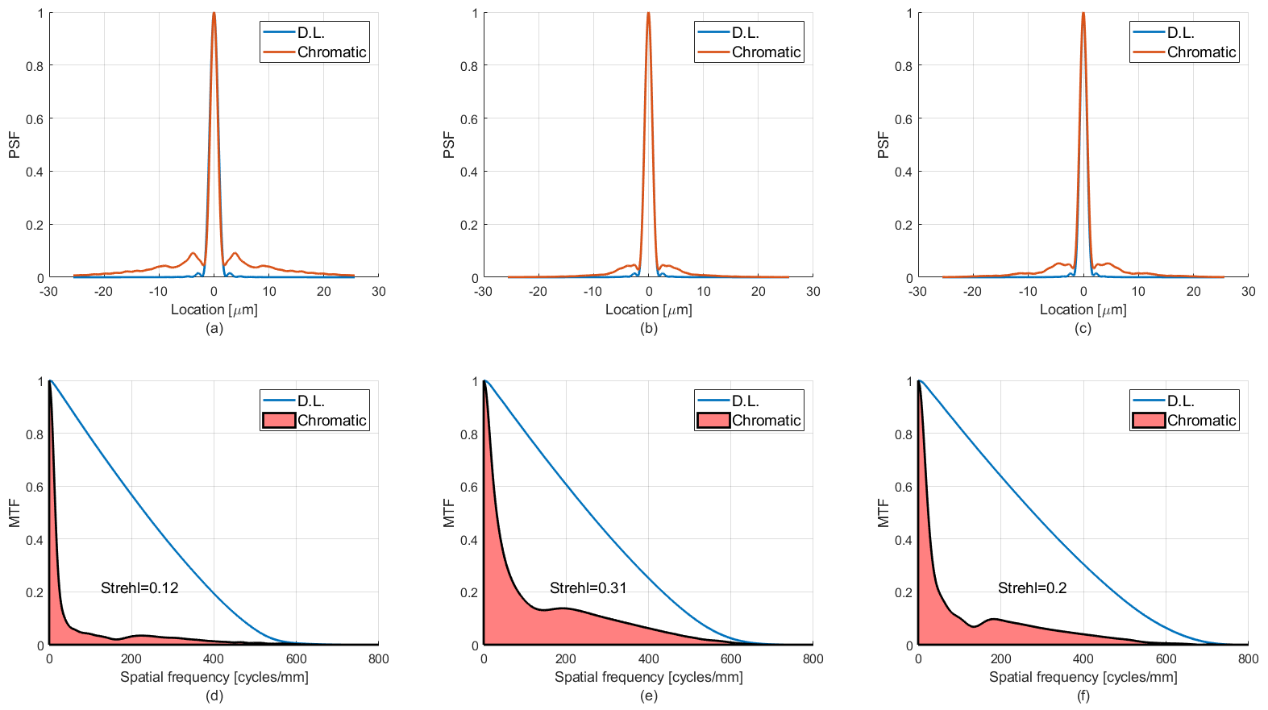


Figure S5: PSFs and MTFs of CDL equivalent to design 2 (NA 0.18) of  [10] over RGB spectral ranges, defined according to spectral sensitivity of Thorlabs DCC1645C color camera. (a) R PSF cross section (b) G PSF cross section (c) B PSF cross section (d) R MTF (e) G MTF (f) B MTF

Table S2. Comparison of ADL of  [10] to equivalent CDL

| Design | 1 | | | | | | 2 | | | | | |
| --- | --- | --- | --- | --- | --- | --- | --- | --- | --- | --- | --- | --- |
| λmin [µm] | 0.45 | | | | | | 0.45 | | | | | |
| λmax [µm] | 0.65 | | | | | | 0.65 | | | | | |
| NA | 0.05 | | | | | | 0.18 | | | | | |
| EFL [µm] | 1000 | | | | | | 1000 | | | | | |
| Dia. [µm] | 100 | | | | | | 366 | | | | | |
| Fresnel no. | 4.6 | | | | | | 60.4 | | | | | |
| Airy rad [µm] | 6.71 | | | | | | 1.86 | | | | | |
| Chr. rad [µm] | 9.1 | | | | | | 33.3 | | | | | |
| DL FWHM | 5.5 | | | | | | 1.5 | | | | | |
|  | **ADL** | | | **CDL** | | | **ADL** | | | **CDL** | | |
|  | **R** | **G** | **B** | **R** | **G** | **B** | **R** | **G** | **B** | **R** | **G** | **B** |
| Efficiency | 0.3 | 0.5 | 0.4 | 0.58 | 0.59 | 0.56 | 0.2 | 0.2 | 0.2 | 0.58 | 0.59 | 0.56 |
| FWHM [µm] | 7 | 6 | 4 | 6.17 | 5.64 | 4.94 | 7 | 7 | 2 | 1.62 | 1.58 | 1.57 |
| 1D Strehl | 1.17 | 1.00 | 0.95 | 0.84 | 0.92 | 0.82 | 0.20 | 0.53 | 0.33 | 0.12 | 0.31 | 0.20 |
| OPM |  |  |  | 0.64 | 0.70 | 0.61 |  |  |  | 0.09 | 0.24 | 0.15 |
| EOPM |  |  |  | 1.45 | 1.78 | 1.7 |  |  |  | 2.75 | 7.93 | 5.49 |
| EOPMcolor |  | | | 1.54 | | |  | | | 3.89 | | |

Since the only resolution data given in [10] is the FWHM, we cannot properly asses the performance of the ADLs. The 1D Strehl ratio values appearing in the table in blue indicate what Strehl ratio the ADL would need to exceed the performance of the CDL. For design 1 the Strehl ratios are near or above 1, meaning that with the reported efficiencies there is no way the ADL can achieve better performance than the equivalent CDL. This is not surprising, since the MTFs of the CDL shown in Fig. S4 are close to the diffraction limit. For design 2, the CDL MTF performance shown in Fig. S5 is quite poor. Therefore, there is much room for the ADL to give improved performance. However, since only FWHM was reported, we cannot know if it does. Again, the 1D Strehl ratios needed for the ADL to exceed the CDL performance are shown in blue. This time they are well below 1, so they can potentially be achieved. However, based on the FWHMs of the ADL, which are larger than those of the CDL, it is not likely that they are achieved (it should be noted that FWHM significantly larger than diffraction limit is usually an indicator of pupil apodization caused by decreased efficiency near the aperture edge, and not of chromatic or spherical aberration, which as shown in Fig. 2 have little effect on the FWHM. The other option is that the aberrations are so large that the Fraunhofer approximation is not valid in the image plane, so we get a Fresnel diffraction pattern which can be broader).

## Spatial multiplexed metalens


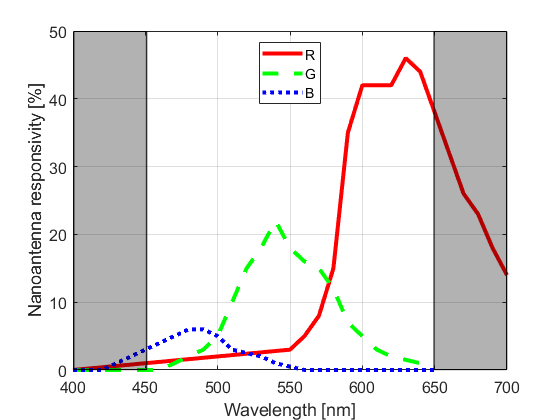


*Figure S6: Spectral response of nanostuctures of*  [32]

## Extended depth of focus metalens

The PSFs of Fig. 7 in the main text are quite similar to the measured results shown in Fig. 2 E-G of [33], but the MTFs are quite different from the calculated results shown in Fig. 2H of [33]. We believe these calculated MTFs are in error, for several reasons: (1) If these MTFs are correct, the EDOF lens gives near diffraction limited resolution. So why is a deconvolution needed to obtain good resolution? (2) In [33] the blue and red images from the EDOF (Fig. 3D) do not look much better than the equivalent images from the conventional metalens (Fig. 3C). However, the red and blue calculated MTFs of the EDOF shown in Fig. 2H are much higher than those of the conventional lens, shown in Fig. 2D. (3) In Fig. 4A of [33], the resolution of the letters R and B looks better for the “Singlet” (conventional) metalens, than for the EDOF metalens. This is in line with our MTF results, which are better for the conventional diffractive lens (Fig. 9) than for the EDOF metalens (Fig. 8). (4) The MTF cutoff stated in [33] in the legend of Fig. 2 is 579c/mm, when in fact it should be $\nu_{c.o.}=\frac{2NA}{\lambda}=\frac{2\cdot0.45}{530e-6}\approx1700c/mm$.

In a more recent publication [34] the authors implemented symmetric EDOFs, which solve the problem of the lateral shift of the PSF (see section 5.4 of main text), and compared their results to conventional metalens results. The reported MTF results seem reasonable. However, in our opinion there is a flaw in the comparison, since it is assumed that the deconvolution kernel must be the same for the R, G and B images, when in fact there is no reason for this. Another difference between our simulation and that of [34] is that we assume a natural scene, so there is overlap in the spectral responses of R, G and B channels. They used an artificial image of an OLED monitor, so there is no spectral overlap. The lack of overlap degrades the conventional metalens resolution.

The SSIM metric used in [33,34] to evaluate overall performance is in our opinion a poor metric, since it lacks physical insight (for example, is the degradation due to noise or to blurring?) and requires a reference image. This metric is more suitable for evaluation of image compression algorithms. The statement made by the authors of [34] that the Strehl ratio is not relevant to their lens, since they perform a deconvolution, is incorrect. The reason deconvolution is useful is because the human brain is much better at averaging noise than it is at performing deconvolutions (if it were good at this, people would not need eyeglasses in high-illumination scenarios, such as outdoors). Deconvolution is not magic. It uses the ability of the computer to perform deconvolutions to improve resolution at the expense of added noise. Therefore, the overall performance described by the metrics defined in this paper does not change as a result of a deconvolution operation [21]. Since most authors, including those of [34], measure MTF and efficiency anyway, all that is left is to measure veiling glare (or total transmission), and one can obtain full performance characterization based on physical parameters, without need for reference image based metrics such as SSIM. These proposed metrics (MTF and SNR) are in fact the industry standard for optical system characterization, and for good reason in our opinion [35,36].

# References

1. J. W. Goodman, *Introduction to Fourier Optics*, 2nd ed. (McGraw-Hill, 1996).

2. D. A. Buralli and G. M. Morris, "Effects of diffraction efficiency on the modulation transfer function of diffractive lenses," Appl. Opt. **31**, 4389 (1992).

3. U. Levy, D. Mendlovic, and E. Marom, "Efficiency analysis of diffractive lenses," J. Opt. Soc. Am. A **18**, 86–93 (2001).

4. A. Arbabi, Y. Horie, A. J. Ball, M. Bagheri, and A. Faraon, "Subwavelength-thick Lenses with High Numerical Apertures and Large Efficiency Based on High Contrast Transmitarrays," Nat. Commun. **6**, 7069 (2015).

5. M. Meem, S. Banerji, C. Pies, T. Oberbiermann, A. Majumder, B. Sensale-Rodriguez, and R. Menon, "Large-area, high-numerical-aperture multi-level diffractive lens via inverse design," Optica **7**, 252–253 (2020).

6. Y. M. Engelberg and S. Ruschin, "Fast method for physical optics propagation of high - numerical - aperture beams," JOSA A **21**, 2135–2145 (2004).

7. D. Faklis and G. M. Morris, "Spectral properties of multiorder diffractive lenses," Appl. Opt. **34**, 2462–2468 (1995).

8. D. W. Sweeney and G. E. Sommargren, "Harmonic diffractive lenses," Appl. Opt. **34**, 2469–2475 (1995).

9. Z. Wang, Y. KIm, and T. D. Milster, "High-harmonic diffractive lens color compensation," Appl. Opt. **60**, 73–82 (2021).

10. N. Mohammad, M. Meem, B. Shen, P. Wang, and R. Menon, "Broadband imaging with one planar diffractive lens," Sci. Rep. **8**, 2799 (2018).

11. M. Meem, S. Banerji, A. Majumder, B. Sensale-Rodriguez, and R. Menon, "Large-area, high-numerical-aperture multi-level diffractive lens via inverse design: reply," Optica **8**, 1011–1012 (2021).

12. J. Engelberg and U. Levy, "Standardizing flat lens characterization," Nat. Photonics **16**, 171–173 (2022).

13. J. Engelberg, C. Zhou, N. Mazurski, J. Bar-David, A. Kristensen, and U. Levy, "Near-IR wide-field-of-view Huygens metalens for outdoor imaging applications," Nanophotonics **9**, 361–370 (2020).

14. J.-C. Diels and W. Rudolph, *Ultrafast Laser Pulse Phenomena*, 2nd ed. (Academic Press, 2006).

15. G. D. Boreman, *Modulation Transfer Function in Optical and Electro-Optical Systems* (SPIE Press, 2001).

16. E. T. F. Rogers, J. Lindberg, T. Roy, S. Savo, J. E. Chad, M. R. Dennis, and N. I. Zheludev, "A super-oscillatory lens optical microscope for subwavelength imaging," Nat. Mater. **11**, 432–435 (2012).

17. V. N. Mahajan, *Optical Imaging and Aberrations, Part II, Wave Diffraction Optics*, Second Ed. (SPIE Press, 2011).

18. L. Levi, *Applied Optics Vol. 1* (John Wiley & Sons, Ltd, 1968).

19. W. J. Smith, *Modern Optical Engineering*, 3rd ed. (McGraw-Hill, 2000).

20. I. Tomić, I. Karlović, and I. Jurič, "Practical assessment of veiling glare in camera lens system," J. Graph. Eng. Des. **5**, 23–28 (2014).

21. S. F. Prokushkin and E. Galil, "Information theoretic methods for image processing algorithm optimization," SPIE Proceedings, Image Qual. Syst. Perform. XII **9396**, 939604 (2015).

22. J. Engelberg, T. Wildes, C. Zhou, N. Mazurski, J. Bar-David, A. Kristensen, and U. Levy, "How good is your metalens? Experimental verification of metalens performance criterion," arXiv:2002.07425 (2020).

23. N. Lindlein, S. Quabis, U. Peschel, and G. Leuchs, "High numerical aperture imaging with different polarization patterns," Opt. Express **15**, 5827–5842 (2007).

24. A. J. E. M. Janssen, S. van Haver, J. J. M. Braat, and P. Dirksen, "Strehl ratio and optimum focus of high-numerical-aperture beams," J. Eur. Opt. Soc. **2**, (2007).

25. G. M. Lerman and U. Levy, "Effect of radial polarization and apodization on spot size under tight focusing conditions," Opt. Express **16**, 4567–4581 (2008).

26. R. Oron, J. L. Guedalia, N. Davidson, A. A. Friesem, and E. Hasman, "Anomaly in a high-numerical-aperture diffractive focusing lens," Opt. Lett. **25**, 439 (2000).

27. U. Levy, E. Marom, and D. Mendlovic, "Thin element approximation for the analysis of blazed gratings: Simplified model and validity limits," Opt. Commun. **229**, 11–21 (2004).

28. G. J. Swanson, "Binary Optics Technology : The Theory and Design of Multi-level Diffractive Optical Elements," Lincon Lab. Tech. Rep. **854**, 1–53 (1989).

29. D. D. O’Shea, T. J. Suleski, A. D. Kathman, and D. W. Praather, *Diffractive Optics* (SPIE Press, 2003).

30. W. T. Chen, A. Y. Zhu, J. Sisler, Z. Bharwani, and F. Capasso, "A broadband achromatic polarization-insensitive metalens consisting of anisotropic nanostructures," Nat. Commun. **10**, 355 (2019).

31. W. T. Chen, A. Y. Zhu, V. Sanjeev, M. Khorasaninejad, Z. Shi, E. Lee, and F. Capasso, "A broadband achromatic metalens for focusing and imaging in the visible," Nat. Nanotechnol. **13**, 220–226 (2018).

32. B. Wang, F. Dong, Q.-T. Li, D. Yang, C. Sun, J. Chen, Z. Song, L. Xu, W. Chu, Y. Xiao, Q. Gong, and Y. Li, "Visible-Frequency Dielectric Metasurfaces for Multiwavelength Achromatic and Highly Dispersive Holograms," Nano Lett. **16**, 5235−5240 (2016).

33. S. Colburn, A. Zhan, and A. Majumdar, "Metasurface optics for full-color computational imaging," Sci. Adv. **4**, eaar2114 (2018).

34. L. Huang, J. Whitehead, S. Colburn, and A. Majumdar, "Design and analysis of extended depth of focus metalenses for achromatic computational imaging," Photonics Res. **8**, 1613 (2020).

35. J. M. Geary, *Introduction to Optical Testing* (SPIE, 2010).

36. G. C. Holst, *Electro-Optical Imaging System Performance*, 5th Ed. (SPIE, 2008).

1. <https://www.thorlabs.com/newgrouppage9.cfm?objectgroup_id=4024&pn=DCC1645C#5316> [↑](#footnote-ref-1)
